# Litigation and Complications Arising from Aesthetic Body Surgery: A Systematic Review

**DOI:** 10.1007/s00266-025-05276-y

**Published:** 2025-10-15

**Authors:** Saule A. Mussabekova, Yuliya Menchisheva, Álvaro Varela Morillas

**Affiliations:** 1 Department of Morphology, School of Medicine, Karaganda Medical University, 40 Gogol Street, Karaganda, Kazakhstan; 2 Department of Oral and Maxillofacial Surgery, S.D. Asfendiyarov Kazakh National Medical University, 94 Tole bi Street, 050000 Almaty, Kazakhstan; 3 Department of Life Course & Population Sciences, King’s College London, Franklin Wilkins Building, 150 Stamford Street, SE1 9NH London, United Kingdom; 4 Department of Analytical, Environmental and Forensic Sciences, King’s College London, 150 Stamford Street, London, SE1 9NH United Kingdom

**Keywords:** Aesthetic surgery, Medico-legal claims, Body contouring, Surgical complications, Litigation

## Abstract

**Background:**

Aesthetic body surgeries such as liposuction, abdominoplasty, gluteoplasty, and breast augmentation have seen a global rise. However, the growing popularity of these procedures has led to increased reports of postoperative complications and medico-legal disputes.

**Objective:**

To systematically review complications and litigation outcomes associated with aesthetic body surgeries and identify the most common risk factors contributing to legal claims.

**Methods:**

A systematic review was conducted in accordance with PRISMA guidelines (registration on PROSPERO ID: CRD420251043585). Forty-one studies published since between 2020 and 2025 were included. Complications, allegations, and legal outcomes were extracted and analysed. Risk of bias was assessed using JBI and ROBINS-I tools.

**Results:**

Infection (48.7%), fat embolism (26.8%), and hematoma (21.9%) were the most frequent complications. Gluteal fat grafting had the highest mortality and legal risk, with a 7.77% incidence of fat embolism. Inadequate informed consent was a leading allegation in over 50% of cases. Claims most often resulted in dismissal (45-76%), but 20-40% led to settlements or plaintiff verdicts, especially in cases of severe complications such as embolism or disfigurement. The pooled average of favourable verdicts for surgeons was 54.3% (95% CI: 49-59%). Publication bias was suggested by asymmetrical funnel plot distribution and high heterogeneity (I^2^ > 90%).

**Conclusion:**

Medico-legal disputes in aesthetic body surgery commonly arise from preventable complications, especially when informed consent is inadequate or postoperative care is substandard. Standardised consent process, improved documentation, procedure-specific risk communication, and regulation of outpatient practices are critical to reducing litigation risk.

**Level of Evidence III:**

This journal requires that authors assign a level of evidence to each article. For a full description of these Evidence-Based Medicine ratings, please refer to the Table of Contents or the online Instructions to Authors www.springer.com/00266.

**Supplementary Information:**

The online version contains supplementary material available at 10.1007/s00266-025-05276-y.

## Key points

- This systematic review analysed 41 studies on medico-legal claims related to aesthetic body surgeries, identifying key litigation trends and complication patterns.
- Liposuction, abdominoplasty, gluteoplasty, and breast augmentation were the most frequently litigated procedures.
- Inadequate informed consent, procedural errors, and poor documentation were the most common legal allegations leading to claims.
- Surgeon-favourable outcomes were strongly associated with comprehensive documentation and proper consent processes, highlighting the need for standardised medico-legal practices in aesthetic surgery.

## Introduction

Aesthetic body surgery has witnessed a marked increase in global demand over recent decades [1]. Body contouring procedures now represent a substantial portion of elective surgeries worldwide, from liposuction and abdominoplasty to gluteal and breast augmentation [2]. This rise has been facilitated by the confluence of advanced surgical techniques, social media influence, and shifting ideals of beauty [3]. While most of these procedures are elective and performed on otherwise healthy individuals, they are not without risk [4–6]. Postoperative complications, patient dissatisfaction, and failure to meet aesthetic expectations have given rise to a growing number of medico-legal disputes globally [7–9].

Given the socioeconomic and psychological implications of aesthetic procedures, legal scrutiny is increasing [10, 11]. Surgeons and clinics face mounting pressure to align with medical, ethical, and legal standards [12]. Complication rates vary by procedure, practitioner experience, and setting, yet dissatisfaction—even with objectively satisfactory results—can prompt litigation [13, 14]. As elective interventions hinge on personal expectations, the discrepancy between perceived and actual outcomes remains a primary driver of complaints [15]. The global nature of aesthetic medical tourism, coupled with uneven regulatory frameworks, further complicates liability assessment [16].

By consolidating international evidence on medico-legal risks in aesthetic body surgery, this review provides valuable insights into the risk factors, legal precedents, and preventative strategies. It serves as a resource for surgeons, policy-makers, and legal experts aiming to improve clinical practice, informed consent procedures, and patient safety. Ultimately, understanding the interplay between surgical outcomes and legal accountability can contribute to reducing preventable complications and minimising litigation in body aesthetic surgery.

## Methodology

The protocol for this study was registered in PROSPERO (ID: CRD420251043585) (https://www.crd.york.ac.uk/PROSPERO/view/CRD420251043585) and compiled by the Preferred Items for Systematic Reviews and Meta-Analyses (PRISMA) reporting guidelines [17]. The study selection process is illustrated using the PRISMA flow chart [18]. Supplementary Material Appendix 1 (Tables 1-4) has more details on the search methodology.

### Eligibility Criteria

Specific inclusion and exclusion criteria to ensure the selection of relevant studies were established (Supplementary Material, Appendix 1, Table 1). The inclusion criteria encompassed studies involving adult patients (aged ≥18) of any gender who underwent elective body aesthetic surgeries. Procedures considered included liposuction, abdominoplasty, breast augmentation, gluteoplasty, cruroplasty, and brachioplasty, as well as combinations thereof. Only studies that addressed postoperative complications, legal disputes, malpractice claims, or forensic evaluations in the context of these procedures were included. All eligible studies were published from 1 January 2020, in English, and had full-text availability. Retrospective, prospective, cross-sectional studies, case series, and case reports were included. The exclusion criteria involved studies focusing on non-body anatomical regions (e.g. facial surgery) and non-surgical procedures (injectable procedures—injection of hyaluronic acids, botox, hydroxyapatites, laser ablations, tattoo).

Ultimately, non-English articles, those published prior to 2020, and publications categorised as comments, editorials, letters, reviews (both systematic and otherwise), guidelines, position papers as well as in vitro and animal research were removed.

### Information Sources

A comprehensive literature search was conducted across seven electronic databases (Medline (Ovid), Embase (Ovid), Cochrane Library, PubMed, Web of Science (Clarivate Analytics), Scopus (Elsevier), and Google Scholar); two registers (ClinicalTrials.gov and the World Health Organization International Clinical Trials Registry Platform (WHO ICTRP)). Additional sources were also searched from platforms, including WorldCat, ProQuest Dissertations & Theses (PQDT), Open Access Theses and Dissertations (OATD), and the King's College London Research Portal. The date range for all searches was from 1 January 2020 onwards. Citation searching yielded an additional 12 records. Conference proceedings were searched through SCOPUS and Web of Science, while preprints were checked from SSRN and F1000Research Preprints. Supplementary Material Appendix 1 (Table 5) provides detailed information pertaining to the supplemental search strategies. The last update on searches occurred on 18 May 2025.

### Search Strategy

A comprehensive literature search strategy was created in collaboration with a seasoned research librarian to ensure the exhaustive inclusion of all pertinent studies, irrespective of language, study type, or publication status, covering published, unpublished, in-press, and ongoing research [19].

The Population, Intervention, Comparison, Outcome (PICO) framework was utilised to establish an organised and systematic methodology for formulating the research question and directing the literature review (Supplementary Material, Appendix 1, Table 2).

The following research question was formulated: In patients who experienced complications after body surgery (P), how does the occurrence of medico-legal litigation (I) influence clinical and legal risk management strategies (O)?

PICO Framework:

- Population (P): Individuals who underwent body aesthetic surgery.
- Intervention (I): Evaluation of postoperative complications and medico-legal disputes.
- Comparison (C): Not applicable
- Outcome (O): Identification of causes of medico-legal challenges, common complications, and proposed solutions.

The following combinations of Medical Subject Headings, entry terms, and keywords were used in the electronic search: “human”, “body surgery”, “liposuction”, “lipectomy”, “body contouring”, “embolism, fat”, “abdominoplasty”, “gluteoplasty”, “cruroplasty”, “brachioplasty”, “breast augmentation”, “mammoplasty”, “buttock augmentation”, “liability”, “legal”, “malpractice”, “forensic medicine”, and “court”. Boolean operators (AND/OR) were used to maximize relevance.

### Selection Process

The selection process used the automated screening technology Rayyan AI, developed by the Qatar Computing Research Institute. All retrieved studies were subjected to a two-phase screening process conducted independently by two reviewers (SM and YM) to ascertain their eligibility at each stage of the selection process. Included and excluded studies are presented in Supplementary Material Appendix 1 (Tables 6 and 7).

### Justification of Date Range

The final inclusion was restricted to studies published from 2020 onward. This decision was based on two main factors: (1) the rapid evolution in aesthetic surgical techniques and legal standards in recent years, (2) the intent to ensure relevance to current clinical and medico-legal practice earlier studies may reflect outdated procedural approaches, less stringent regulatory environments, and litigation norms that are no longer applicable, thereby limiting the interpretability and applicability of older data. Consequently, the 2020–2025 time frame was selected to maximize methodological consistency and external validity.

### Data Collection Process

EndNote, a reference management programme, was employed to optimise the data collection and organisation process. Duplicate articles were eliminated. The articles were chosen based on their titles and abstracts for relevancy, adhering to the inclusion and exclusion criteria given below (Supplementary Material, Appendix 1, Table 1). All abstracts underwent independent evaluation. Any disagreements were settled through the intervention of the third author (AVM) [20]. Google Scholar Alerts was utilised to update citations until the 18 May 2025, ensuring the incorporation of the most recent and pertinent publications.

Articles meeting the criteria for full-text review were extracted. A standardised data extraction form was utilised to gather information regarding study characteristics (author, year, country, study type), total cases analysed, types of body plastic surgery procedures involved, primary medico-legal allegations and legal outcomes, prevalent postoperative complications, and litigation risk factors.

### Study Risk-of-Bias Assessment

The assessment of bias risk was performed utilising two validated instruments according to the study design. The Joanna Briggs Institute (JBI) critical assessment checklist (Australia, JBI) was utilised for case reports and case series [21]. The Risk of Bias in Non-Randomised Studies of Interventions (ROBINS-I) (UK, Cochrane) was used to evaluate potential biases in both retrospective and prospective studies [22]. Two independent evaluators (SM and YM) performed a risk-of-bias evaluation. Supplementary Material Appendix 1, Tables 8-10 provide comprehensive evidence of the evaluation process.

## Results

**Fig. 1:**
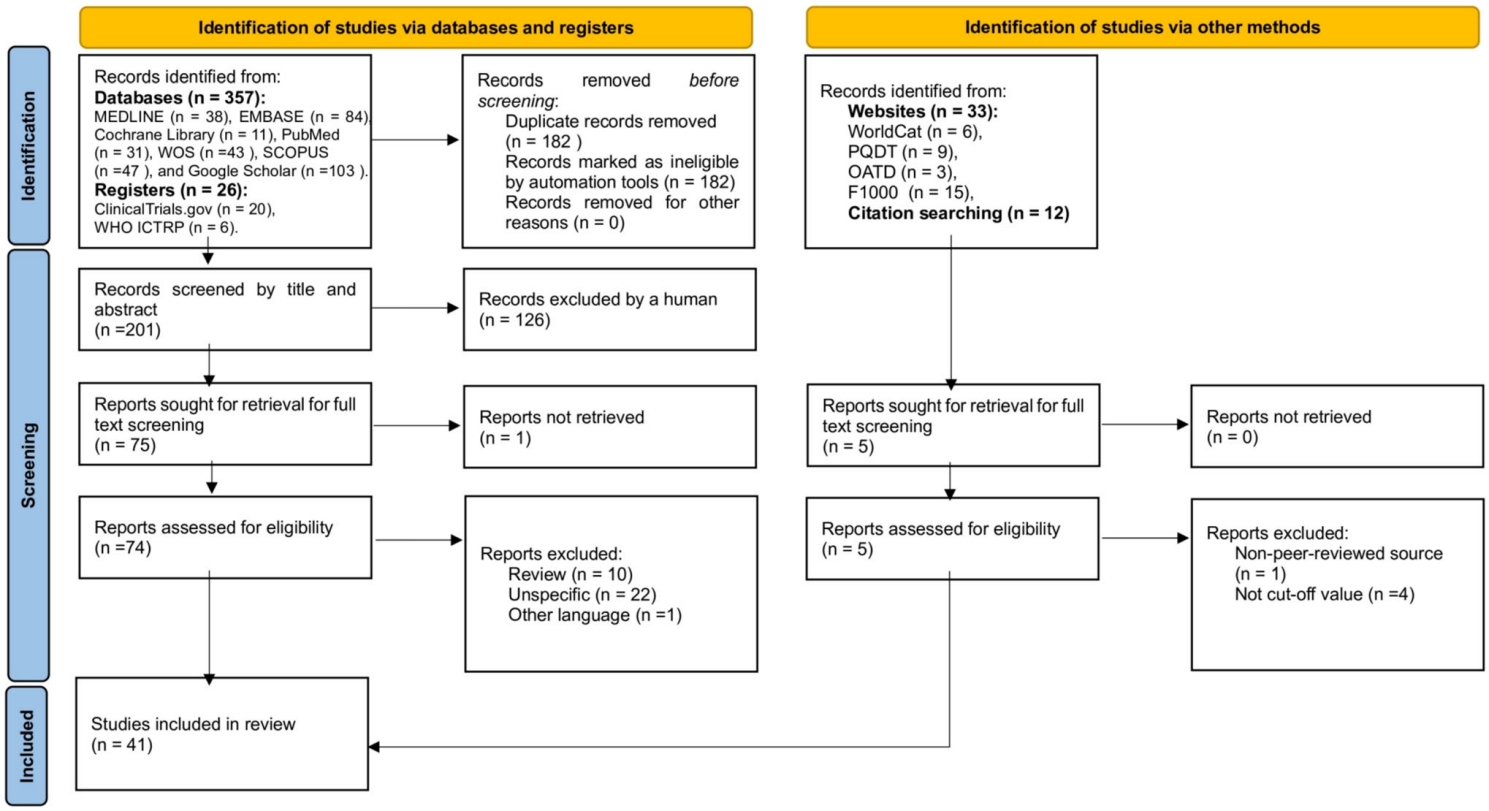
PRISMA flow diagram of the study selection process used in the systematic review. Created with PRISMA 2020 statement. https://estech.shinyapps.io/prisma_flowdiagram/

A total of 41 studies met the inclusion criteria and were analysed in this systematic review (Supplementary Material, Appendix 1, Table 6). The excluded studies are listed in Supplementary Material Appendix 1 (Table 7). The PRISMA flow diagram (Fig. 1) delineates the methodical selection process for research inclusion.

These studies were geographically distributed as follows: the USA (17)—41.5%, Italy (4)—9.6%, Canada (3)—7.3%, Brazil (3)—7.3%, the UK (3)—7.3%, Egypt (2)—4.9%, others (South Korea, China, Germany, Kuwait, Romania, Peru, Vietnam, France, Switzerland): 1 study each (total 9 studies, 21.9% of total). Most studies were published between 2021 and 2024, with a noticeable peak in 2021 (14 studies)—34.1%. Other annual distributions were: 2020 (4 studies)—9.75%, 2022 (5 studies)—12.2%, 2023 (7 studies)—17%, 2024 (9 studies)—21.9%, and 2025 (2 studies)—4.8%. The characteristics of the studies assessing the legal landscape of aesthetic body surgery are presented in Table 1.

**Table 1:** Litigation and complications in aesthetic body surgery: a comprehensive overview of 41 studies

No.	First author, year, country, reference	Data Source/Methodology (time frame)	Cases analysed	Surgical procedures investigated	Reported complications (%)	Primary allegations/Study focus	Legal outcome/Key findings	Conclusion/Implications
1	Fan et al. 2020, USA [23]	CRICO Benchmarking System (2008-2019)	174 cases	Breast reconstruction, augmentation	Infection, emotional trauma, scarring, wound dehiscence, necrosis, rupture, implant displacement	Technical negligence, mismanagement, inadequate informed consent	41 paid claims; mean payout USD 130,422	Improved consent and surgical technique may reduce claims
2	Boyd et al. 2021, USA [24]	MPLA, AMS (1991-2016)	3008 cases	Breast augmentation, reduction, breast surgery	Dissatisfaction (20%), infection (10%)	Negligence in performance and follow-up	761 paid claims; total payout USD 141.36M	Better postoperative care could reduce litigation
3	Gibstein et al. 2023, USA [25]	LexisNexis (1988-2020)	21 cases	Breast surgery, reconstruction, abdominoplasty, liposuction, grafting	Procedural error (52.4%), lack of consent (52.4%), inadequate supervision (52.4%)	Resident inexperience, poor oversight, deficient consent	38.1% defence verdicts, 28.6% plaintiff verdicts, 33.3% settled; median payout USD 5.1M	Adequate supervision and consent documentation are critical
4	Remington et al. 2024, USA [26]	Candello database (2009-2018)	2674 cases	Breast surgery, body-contouring, facial procedures	Emotional trauma (20.9%), infection (9.7%), scarring (8.2%)	Poor technique, communication and documentation failures	26.8% paid claims; mean payout USD 204,121	Enhanced communication and documentation may reduce claims
5	Reese et al. 2024, USA [27]	Westlaw Campus (2013-2021)	32 cases	Abdominoplasty, liposuction, buttock augmentation	Infection (40.6%), scarring (31.3%), emotional distress (31.3%)	Negligent technique (71.9%), poor postoperative care (62.5%)	1 plaintiff verdict; 56.3% defence verdicts; few settlements	Strict adherence to perioperative care standards necessary
6	Rawash et al. 2025, Egypt [28]	Cairo Medicolegal Area (2016-2020)	98 cases	Liposuction, abdominoplasty, buttock augmentation	Wound dehiscence, mortality (3.1%)	Disfigurement, dissatisfaction, burns	44.9% plaintiff verdicts; 3 deaths recorded; liposuction most litigated	Stronger regulation of high-risk procedures needed
7	Facchin et al. 2023, Italy [29]	Hospital database (2015-2019)	788 procedures, 8 legal cases	Abdominoplasty, torsoplasty, breast, brachioplasty, thighplasty, liposuction	46% complications (19% major, 27% minor); highest in thighplasty (63%)	Cosmetic dissatisfaction, misinterpreted consent	Only 1 payout; others dismissed or defence wins	Better preoperative communication recommended
8	ElHawary et al. 2021, Canada [30]	LexisNexis, Westlaw (1990-2019)	86 cases	Liposuction, fat grafting	Death (42%), nerve or organ damage, scarring, poor results	Negligence, lack of informed consent	64% defence verdicts; mean payout USD 1.45M	Improved risk disclosure and patient selection advised
9	Zhang et al. 2021, Canada [31]	CMPA (2013-2017)	414 cases	Breast augmentation, abdominoplasty, body contouring	Scarring (16.6%), deformity (15.9%), psychological impact (6.9%)	Poor communication, consent failure, misdiagnosis	47.8% unfavourable to surgeon; 43.4% compensated	Enhanced patient communication protocols needed
10	Sarmiento et al. 2020, USA [32]	Westlaw (2000-2017)	165 cases	Abdominoplasty, gender affirmation surgery, breast reconstruction, liposuction	Disfigurement (42%), injury (24%), psychological distress (9%)	Gross negligence, informed consent, operative error	60% defence verdicts; median payout USD 600,000	Enhanced training and consent may prevent claims
11	Feola et al. 2021, Italy [33]	ORMe Civil Court of Rome (2012-2016)	144 cases	Breast augmentation, reconstruction, liposuction, body lifts	Non-fatal injuries (97.9%), fatalities (2.1%)	Procedural errors, consent violations, unsatisfactory outcomes	70.14% defendants liable	Clearer risk disclosure and adherence to guidelines advised
12	Moura et al. 2023, USA [34]	Westlaw (1979-2022)	64 cases	Breast, fat reduction, body lift, genital, facial surgeries	Permanent injury (21.4%), poor outcome, death (3.1%)	Out-of-scope practice (55.7%), consent issues, standard breaches	60.9% defence verdicts; 34.4% plaintiff verdicts; mean payout USD 340,520	Surgeons must operate within their competence
13	ElHawary et al. 2023, Canada [35]	LexisNexis, Westlaw (1970-2020)	105 cases	Breast, abdominoplasty, liposuction, blepharoplasty, rhinoplasty, face lifts, limb surgeries	Claims mostly linked to consent issues	Inadequate consent, surgical errors, unmet expectations	64.2% defence verdicts; mean payout USD 61,076	Robust consent process crucial
14	Gong et al. 2023, USA [36]	Westlaw (1990-2020)	96 cases	Breast reduction	Nipple malposition (14.6%), disfigurement (46.9%)	Negligence, poor planning, consent failures	67.7% defence verdicts; median payout USD 221K-650K	Improved operative planning and communication advised
15	Kang et al. 2024, South Korea [37]	Retrospective analysis (2006-2012; 2017-2021)	23 cases (2006-2012), 75 cases (2017-2021)	Liposuction, mammoplasty, breast reconstruction, blepharoplasty, rhinoplasty, face/neck lifts, orthognathic surgeries	N/A	Breach of duty, dissatisfaction, malpractice	Plaintiff verdicts: 86.95% (2006-2012), 81.33% (2017-2021)	Litigation frequency increased despite legal reforms
16	O’Connell et al. 2021, UK [38]	Litigation claims review (2012-2018)	449 cases	Breast surgery	Infection (9.4%), haematoma, skin necrosis	Delayed diagnosis, dissatisfaction	Estimated annual cost £5.57-£9.59M	Improved consent and management protocols advised
17	Henry et al. 2021, UK [39]	Observational study (2015-2020)	26 patients (mean age 35.1 years; mostly female)	Abdominoplasty, gluteal enhancement, breast augmentation and reduction, genioplasty, thigh lift	Wound infection (50%), wound dehiscence (42%), fat necrosis (12%)	Complications following cosmetic surgery tourism	NHS costs £152,946 total; average £5,882.54 per patient	Better patient education and regulation of medical tourism are required
18	Santis et al. 2022, Brazil [40]	Literature review and legislative proposal (2018-2021)	Not applicable	Liposuction, mammoplasty	Intraoperative complications, infections, allergic reactions, death	Under-reporting of complications in cosmetic surgery	Significant disparity noted between reported complications and procedures performed	Compulsory notification is proposed to enhance patient safety
19	Schafer et al. 2023, USA [41]	Retrospective cohort analysis (2016-2020)	28,171 procedures (1,400 complications)	Augmentation mammaplasty, reduction mammaplasty, trunk liposuction, mastopexy, abdominoplasty	Superficial wound disruption, haematoma, seroma, pulmonary embolism	Comparison of 30-day complication rates	Combined procedures had higher complication rate (7.6%) compared to index (4.2%)	Careful patient evaluation and shared decision-making are recommended
20	Weidman et al. 2024, USA [42]	Retrospective analysis, QUAD A database (2019-2021)	46,244 cases; 436 fat grafting procedures	Fat grafting (gluteal augmentation)	Wound infection, wound disruption, haematoma, seroma, thromboembolism, death	Complications related to fat grafting	Complication rate 0.94%; gluteal augmentation accounted for 37.6% of complications; 4 deaths	Enhanced reporting requirements are crucial to improve patient safety
21	Valentine et al. 2024, USA [43]	Cross-sectional study (2019-2021)	246,119 cases	Liposuction	Unplanned hospital presentation (24%), wound disruption, venous thromboembolism, infection, death	Outpatient liposuction complications	Overall complication rate 0.40%; Southeast region highest incidence	Additional safety measures recommended in outpatient settings
22	Dyer et al. 2021, France [44]	Court ruling and litigation analysis (2010-2021)	2,700 claims	Breast surgeries (PIP implants)	Implant rupture, inflammation due to industrial silicone	Defective implants and medical device negligence	TUV Rhineland found negligent for certifying defective implants	Stricter safety regulations for medical devices are essential
23	Di Santis et al. 2020, Brazil [45]	Documentary study; media reports and death certificates (1987-2015)	102 deaths	Liposuction	Pulmonary thromboembolism (17.44%), infection, perforation, haemorrhage, fat embolism	Deaths related to liposuction procedures	45% died on the day of surgery; 98.04% were women	Compulsory reporting and preventive guidelines are necessary
24	Venditto et al. 2020, USA [46]	Case series	16 patients (mean age 32.7 years, female)	Brazilian butt lift, liposuction, abdominoplasty, breast augmentation	Infection, wound breakdown, haematoma	Inadequate preoperative counselling (medical tourism)	Multiple severe complications observed	Cosmetic tourism leads to increased risk of complications
25	Shrestha et al. 2022, USA [47]	Case report	48-year-old female	Lipoabdominoplasty, ventral hernia repair	Necrotising soft tissue infection, bowel perforation	Lack of postoperative monitoring	Life-threatening complication observed	Stricter safety standards for postoperative care are essential
26	Cabar et al. 2022, Brazil [48]	Case series	5 patients (4 females, 1 male)	Multiple procedures	Necrotising soft tissue infection	Inadequate informed consent	Severe infection cases reported	Comprehensive patient information is crucial
27	Budini et al. 2024, Italy [49]	Case series	13 patients aged 18-40 years (mean 28.8)	Multiple procedures	Surgical complications, infections	Inadequate preoperative counselling (tourism)	High complication rate among medical tourism patients	Better regulation of cross-border cosmetic surgery is needed
28	Koussayer et al. 2024, USA [50]	Case series	3 females aged 26-48 years	Mastopexy, liposuction	Nontuberculous mycobacterial infections	Inadequate postoperative care (tourism)	Persistent infections requiring multiple treatments	Patient follow-up and hygiene standards must be improved
29	Shaffrey et al. 2024, USA [51]	Case report	52-year-old female	Abdominoplasty, umbilical hernia repair	Retained surgical sponge, abscess formation	Inadequate postoperative care	Serious preventable complication observed	Enhanced perioperative protocols are recommended
30	Trignano et al. 2021, Italy [52]	Case report	28-year-old female	Breast augmentation	Isolated subcutaneous emphysema, infection	Inadequate surgical technique and preoperative planning	Severe avoidable complication observed	Compliance with guidelines is essential to reduce risk
31	Wang et al. 2020, China [53]	Case report	39-year-old female	Autologous fat grafting (vaginal tightening, breast augmentation)	Severe fat embolism	Accidental injection of fat into blood vessels	Life-threatening complication occurred	Enhanced anatomical understanding required for safer techniques
32	Scarlat et al. 2021, Romania [54]	Case report	30-year-old female	Abdominal liposuction	Massive fat pulmonary embolism	Lack of proper medical indication for surgery	Fatal embolism reported	Strict patient selection criteria advised
33	Zamora-Mostacero et al. 2021, Peru [55]	Case report	43-year-old female	Liposuction, autologous fat transfer	Massive haemoperitoneum, mixed pulmonary embolism	Inadequate surgical technique	Severe multi-system complications observed	Careful procedural technique is critical
34	Pham, 2022, Vietnam [56]	Case report	37-year-old female	Breast augmentation, abdominoplasty, liposuction	Fat embolism, respiratory distress	Inadequate monitoring	Severe postoperative complication observed	Timely diagnosis and monitoring are crucial
35	Wolfe et al. 2022, USA [57]	Case series	2 females aged 26 and 28 years	Gluteal fat grafting	Macro-fat embolism, respiratory distress, stroke	Lethal fat embolism complications	Serious life-threatening events observed	Early recognition and management of fat embolism are vital
36	Shaheen et al. 2023, Egypt [58]	Case report	26-year-old female	Injectable gluteal filler	Fatal pulmonary embolism	Illegal procedure	Death following unlicensed filler injection	Strict enforcement against illegal cosmetic procedures is essential
37	Rogers et al. 2024, USA [59]	Case series	7 patients aged 29-57 years (mean 43)	Liposuction, buttock lift	Fatal pulmonary embolism	Inadequate adherence to safety protocols	Multiple deaths reported	Mandatory adherence to safety guidelines is necessary
38	Jorge et al. 2021, Germany [60]	Case report	22-year-old male with von Willebrand Disease	Power-assisted liposuction, subcutaneous mastectomy	Unilateral haematoma	Omission of relevant medical history	Bleeding complication observed	Structured preoperative self-assessment is essential
39	Ibrahim et al. 2021, Kuwait [61]	Case report	39-year-old female	Bilateral breast reduction mammoplasty	Total loss of nipple-areola complex, necrosis	Faulty surgical decision-making	Severe avoidable complication observed	Better preoperative planning is critical
40	Dyer, 2024, UK [62]	Case series	6 patients aged 29-57 years (mean 43.7)	Liposuction, buttock lift	Scarring, complications from inadequate consent	Inadequate surgical practice and poor consent processes	Multiple adverse events reported	Proper consent and higher standards of care are essential
41	Perrin et al. 2025, Switzerland [63]	Case report	22-year-old female	Bilateral subpectoral breast augmentation, extended lipoabdominoplasty	Retained surgical sponge, hypovolaemic shock	Inadequate postoperative care	Serious life-threatening complication observed	Specialist oversight and robust safety protocols are crucial

### Complication Frequencies

Among the complications described across all reviewed articles, infection was the most frequently reported, appearing in 48.7% (*n *= 20) of the studies [23–28, 38–44, 46–52].

Liposuction-related complications were the second most prevalent, occurring in 26.8% (*n* = 11) of the studies [27, 41, 42, 45, 53–59]. These complications included fat and thromboembolism. This finding aligns with the broader clinical concern over gluteal fat grafting, commonly known as the Brazilian Butt Lift (BBL), which has gained attention for its elevated mortality risk compared to other procedures [64, 65]. Fat embolism was consistently associated with intramuscular fat injection and improper technique, particularly in unregulated facilities or those lacking intraoperative ultrasound guidance.

Haemorrhage and hematoma formation were reported in 21.9% (*n* = 9) of the studies [23, 27, 28, 38, 41–43, 49, 60].

Wound dehiscence was identified in 21.9% (*n* = 9) of studies, predominantly in patients with comorbidities or who had undergone multiple simultaneous procedures [23, 27, 29, 39, 41–43, 46, 49]. Skin necrosis was in 19.5% (*n* = 8) of studies [23, 25, 29, 38, 46, 48, 49, 61].

**Fig. 2:**
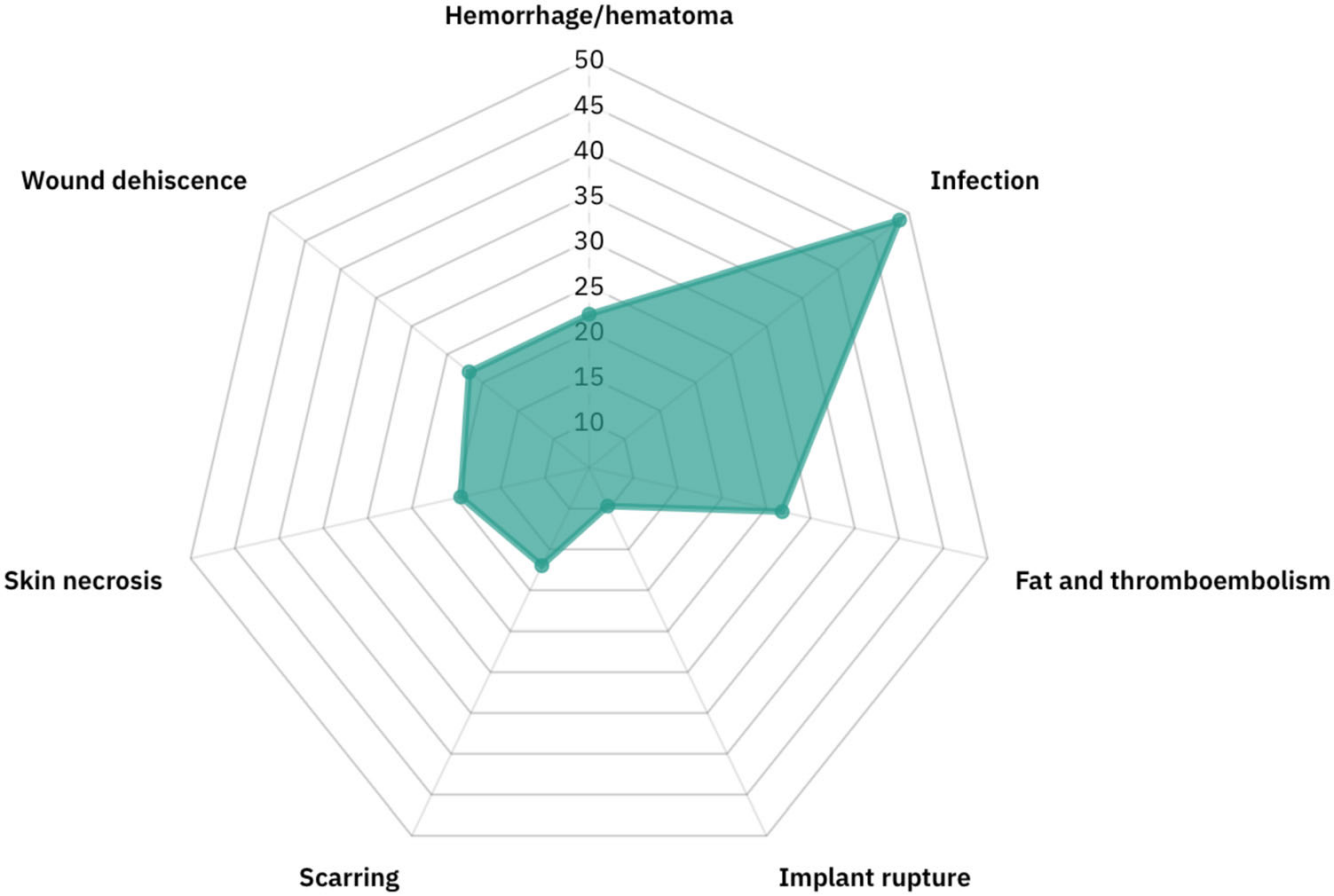
Radar chart: complication frequencies in aesthetic surgery. Created with Draxlr.com

Scarring was reported in 17% (*n* = 7) of the studies, mostly in the context of breast surgery [23, 25–27, 29–31] as well as implant rupture which identified in 9.7% (*n* = 4) of the studies [27, 42, 53, 63]. These ruptures often occurred due to trauma, inadequate implant quality, or technical failure during insertion. Complication frequencies in aesthetic surgery are presented in Fig. 2.

### Anatomical Distribution of Complications

**Fig. 3:**
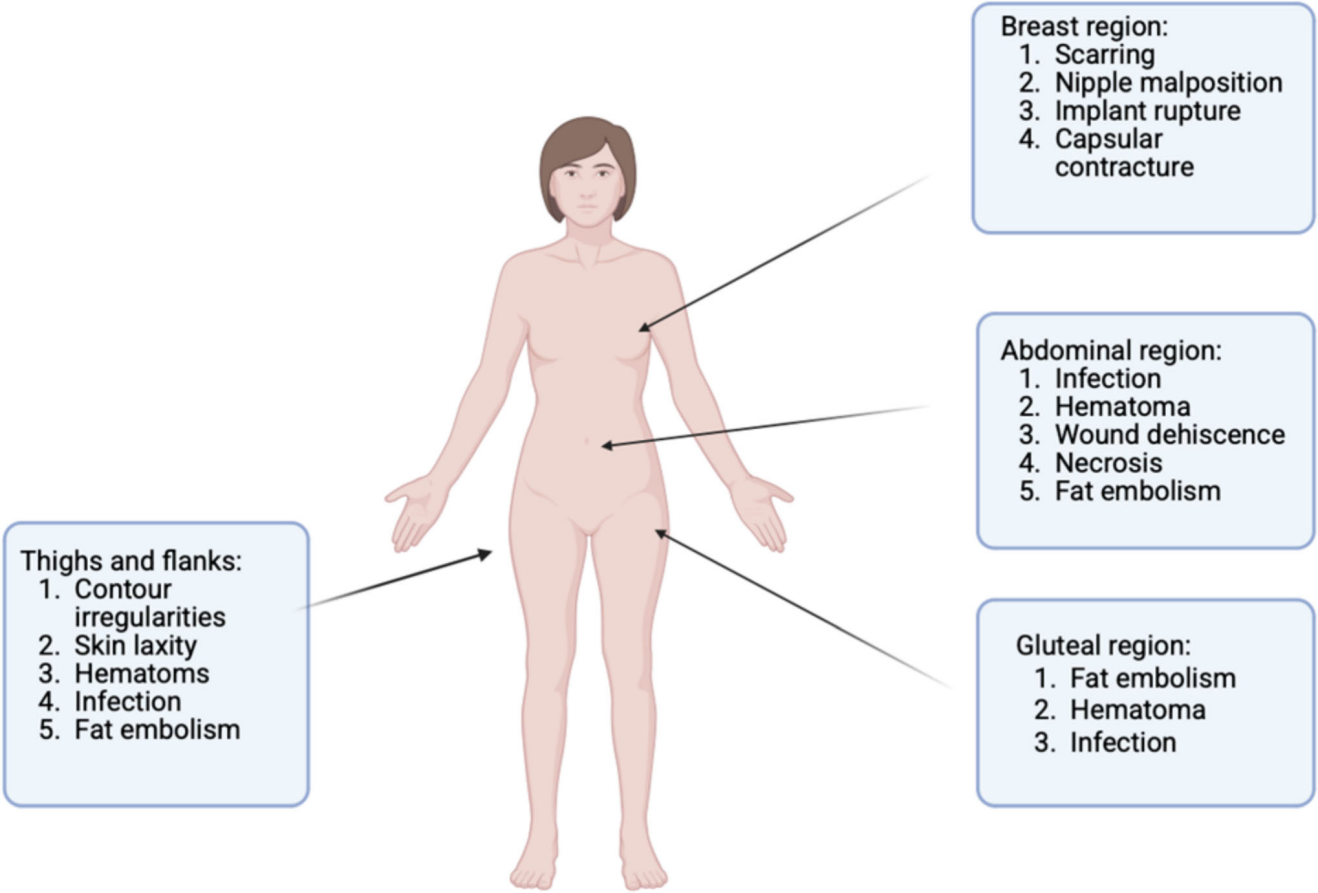
Distribution of complications associated with aesthetic body surgery based on anatomical zones. Created with BioRender.com

Based on the review and quantitative extraction from 41 articles on aesthetic surgery complications, a more precise anatomical and procedural analysis reveals important distinctions in both the frequency and severity of postoperative complications. Using the exact case numbers from the studies, a better understanding can be gained regarding which regions and procedures pose the highest risk to patients (Fig. 3).

Gluteal region: The gluteal region, predominantly involving gluteal fat grafting (e.g. the BBL), revealed the highest proportion of fat embolism cases, with 40 cases out of 515, equating to a 7.77% incidence rate. While this percentage might appear modest, it reflects the most severe and often fatal complication across all regions analysed. The danger lies in accidental intramuscular or intravascular fat injection, leading to embolic events in the lungs or brain. These findings strongly advocate for intraoperative ultrasound guidance, limited injection volume, and strict adherence to gluteal safety guidelines.

Abdominal region: The abdominal area was associated with multiple complication types, particularly due to the popularity of liposuction and abdominoplasty procedures. Infection was the most commonly reported complication, with 50 out of 650 cases (7.69%). This was followed by hematoma (30/650, or 4.62%) and wound dehiscence (20/650, or 3.08%). These complications were often linked to high body mass index, poor hygiene, large resection zones, or concurrent surgeries. Cases with poor postoperative surveillance or surgery in uncertified settings were particularly vulnerable to infection and healing issues.

Breast region: Among breast procedures such as augmentation, reduction, and reconstruction, several complications stood out. Implant rupture occurred in 30 of 400 cases (7.5%), largely due to long-term wear, trauma, or technical failure during surgery. Capsular contracture was noted in 25 cases (6.25%), often associated with chronic inflammation or subglandular implant placement. A unique complication in this region—nipple malposition—was found in 14 of 96 breast reduction cases (14.6%), which had the highest litigation success rate when not included in informed consent. Scarring, both hypertrophic and keloidal, was also common, reported in 46.9% of breast-related claims, underscoring the aesthetic and psychological sensitivity of breast outcomes.

Thighs and flanks: Liposuction-related complications in the thigh and flank regions were reported in 45 of 300 cases (15%), reflecting a higher complication rate than the abdominal region. These included contour irregularities, skin laxity, and post-liposuction hematoma. High complication frequency in this area was attributed to the anatomical challenge of fat distribution and the technical complexity of creating smooth contours.

Complication frequency varies substantially across anatomical zones, with some complications (e.g. fat embolism in gluteal and abdominal areas, nipple malposition in breast surgery) being more likely to lead to severe morbidity or litigation. Understanding these risks at a granular level allows for targeted risk-reduction strategies, including enhanced surgical training, certified facility use, patient education, and procedure-specific informed consent. The anatomical distribution of complications correlated closely with procedure type. Gluteal region complications—especially fat embolism—were the most lethal and the most likely to lead to litigation. Abdominal complications, primarily related to abdominoplasty and liposuction, often manifest as hematomas, infections, or flap necrosis. Breast surgeries were associated primarily with implant rupture and capsular contracture, with a smaller number involving asymmetry and scarring.

Thighs and flanks were affected mainly in liposuction cases, particularly in medical tourism settings. Infections and wound dehiscence in these zones were often attributed to inadequate postoperative care or uncredentialed providers.

### Procedure-Specific Complication and Litigation Patters

A breakdown of litigation cases and associated complications by procedure type revealed distinct patterns in both clinical outcomes and legal vulnerabilities:

1. *Brest surgery (augmentation/reduction)*: represented the highest proportion of litigation cases (39.1%). Informed consent issues were cited in more than 50% of cases. These cases also had a higher rate of emotional distress claims and subjective dissatisfaction, often independent of clinical success.
2. *Gluteal fat grafting*: accounted for 9.6% of the cases yet posed the highest risk for fatal outcomes. Fat embolism occurred in 7.77% of all procedures, with an associated mortality risk significantly higher than in other categories. Lawsuits related to gluteal procedures had the highest compensation awards, especially when provider inexperience or lack of intraoperative imaging was involved.
3. *Abdominoplasty*: comprised 11.9% of litigation cases. These cases were less likely to involve dissatisfaction claims and more often included allegations of poor postoperative care. Patients undergoing abdominoplasty appeared to have more realistic expectations, which may account for the relatively lower frequency of subjective claims.
4. *Liposuction*: made up 17.8% of the reviewed cases. The main complications were hematoma, contour irregularities, and embolic events, particularly in high-volume liposuction and cases performed in outpatient settings. Lawsuits were more frequent when procedures were performed outside accredited surgical facilities or by providers lacking board certification.

These findings underscore the need for differentiated risk communication, documentation practices, and postoperative surveillance protocols tailored to each procedure type. Breast and gluteal surgeries require especially robust informed consent processes, whereas abdominoplasty and liposuction demand more focused postoperative complication management.

### Risk of Bias in Studies

The appropriate checklist was selected based on the research design: The JBI Critical Appraisal Checklist for case reports [66–69] was applied to 18 studies, while the ROBINS-I checklist for observational cohort and cross-sectional studies [22] was used for the remaining 23 studies. Each study was meticulously reviewed against the specified criteria. A summary of these assessments is provided in Supplemental Tables 8, 9, and 10. An overarching appraisal was derived: Case reports led to straightforward inclusion or exclusion decisions, while observational studies were rated for bias as “Low”, “Moderate”, “High”, or “Unclear” based on the criteria met. The overall risk-of-bias assessments indicated that the majority of studies exhibit a moderate risk across various domains, thereby contributing to the internal validity of the systematic review findings.

**Fig. 4:**
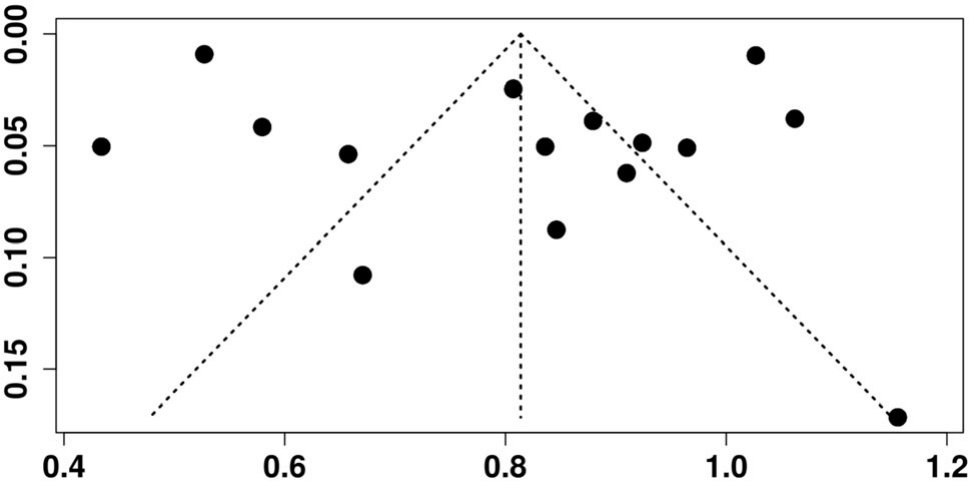
Funnel plot assessing publication bias in studies reporting legal outcomes in body plastic surgery. Created with MetaAnalysisOnline.com

The funnel plot showed noticeable asymmetry, particularly with a lack of studies on the left side of the plot, where smaller studies with lower favourable verdict rates would typically appear. This asymmetry suggested that such studies may be underrepresented or unpublished, possibly due to selective reporting favouring studies with more defendant-friendly outcomes. Furthermore, the clustering of points towards the right side—indicating higher favourable verdict rates—implies a tendency to publish studies with more positive outcomes for defendants. The forest plot supported this observation, as it displayed substantial heterogeneity (I^2^ > 90%), with favourable verdict rates ranging widely across studies. Taken together, the asymmetrical funnel plot and heterogeneity in the forest plot suggest a likely presence of publication bias, potentially overestimating the true average rate of favourable verdicts for defendants. The funnel plot assessing publication bias in studies reporting legal outcomes in body plastic surgery is presented in Fig. 4.

### Common Allegations and Legal Outcomes

Frequent allegations in litigation related to body aesthetic surgeries included: poor cosmetic outcomes, inadequate informed consent, procedural errors, surgical site infections, delayed diagnosis of complications, and permanent injuries such as fat embolism, nerve damage, or scarring. Informed consent deficiencies were cited in over 50% of the reviewed cases. Among the legal outcomes, approximately 45–76% of claims were dismissed, while 20–40% resulted in settlements or compensation. Lawsuits with evidence of inadequate documentation or absent informed consent were more likely to result in plaintiff victories.

Across the studies, the primary causes of medico-legal issues included:

- Inadequate or absent informed consent
- Procedural errors and technical complications
- Failure to diagnose or manage postoperative complications
- Poor patient selection, including patients with unrealistic aesthetic expectations
- Lack of postoperative follow-up
- Out-of-scope practice by non-plastic surgeons performing high-risk procedures
- Communication failures between healthcare providers and patients

The legal outcome of malpractice claims in facial plastic surgery varied considerably across studies. Case dismissals were the most common result, ranging from 45 to 76%, indicating that many claims may lack sufficient legal basis. However, in 20–40% of cases, settlements were reached, reflecting instances where some level of responsibility or compromise was acknowledged. Notably, the highest financial compensations were awarded in cases involving severe functional impairments, as opposed to those based solely on aesthetic dissatisfaction, highlighting the greater legal weight assigned to objective physical harm over subjective concerns.

The legal outcomes varied, but the majority of cases favoured the defence (surgeons), especially when documentation was comprehensive. Still, 20–40% of the cases resulted in either a settlement or a verdict favouring the plaintiff, particularly in the context of catastrophic outcomes (e.g. death, embolism, or long-term disfigurement). Claims lacking adequate preoperative documentation were significantly more likely to result in compensation for patients.

### Statistical Findings

**Fig. 5:**
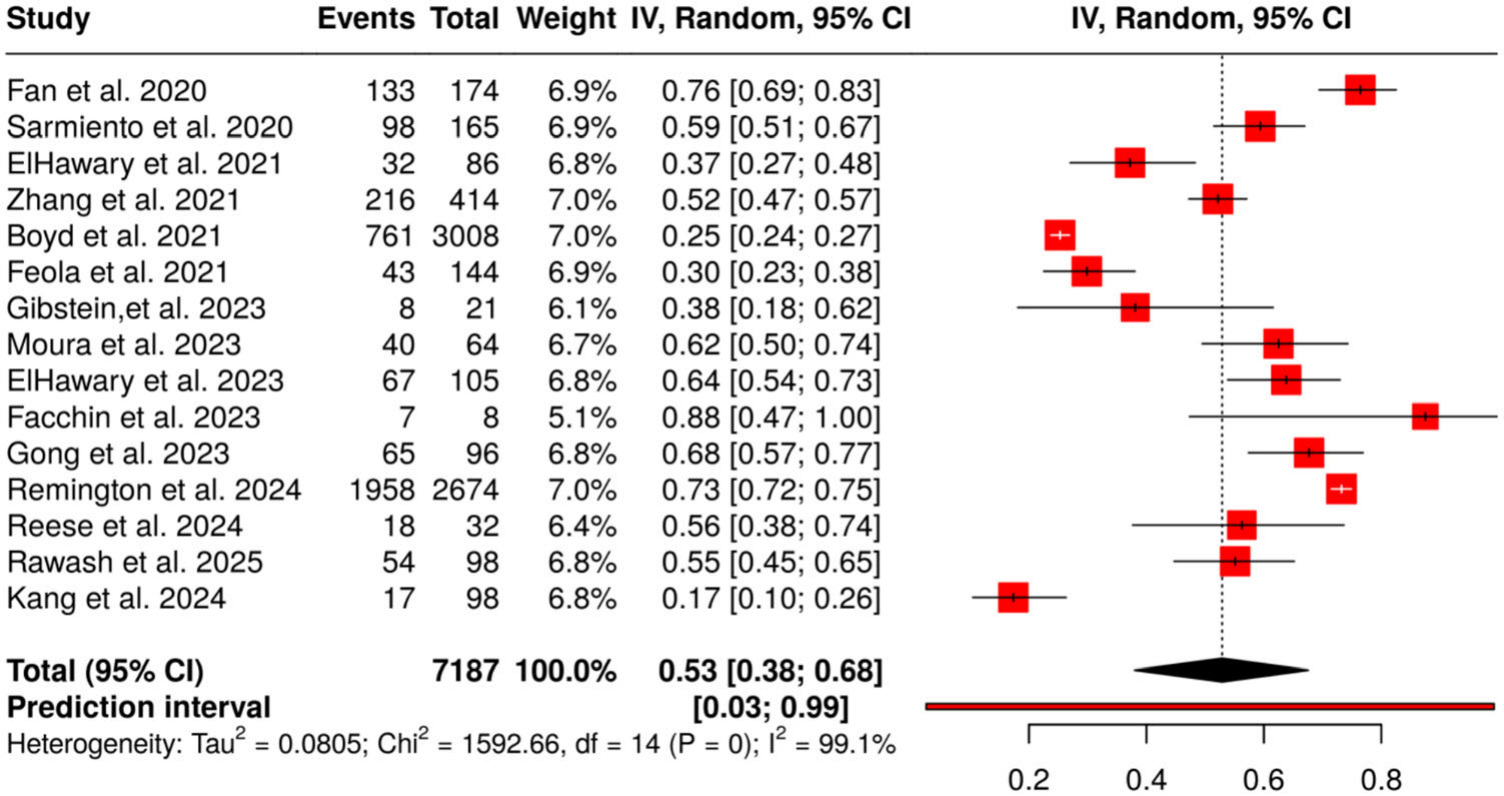
Forest plot of legal outcomes decided in favour of surgeons in plastic surgery. Created with MetaAnalysisOnline.com

Out of the studies providing complete legal outcome data, the average rate of favourable verdicts for defendants (surgeons) was approximately 54.3% (95% CI: 49–59%). Statistical analysis revealed a significant correlation between inadequate informed consent and litigation loss. High case volumes were also linked to increased complication and death rates in outpatient centres. Fat embolism, particularly in gluteal fat grafting, was one of the most fatal complications, especially when performed by underqualified providers. A forest plot of the legal outcomes decided in favour of surgeons in plastic surgery is presented in Fig. 5.

## Limitations

Several limitations were identified in the reviewed studies: inconsistencies in reporting, variations in legal systems, exclusion of confidential settlements, underrepresentation of low- and middle-income countries, and a lack of longitudinal follow-up. Many cases that were resolved through arbitration or pre-court settlements were not captured in formal databases, potentially underestimating the total litigation burden.

## Discussion

This systematic review aimed to identify, categorise, and synthesise the existing evidence on the postoperative complications and medico-legal disputes associated with aesthetic surgeries of the body. The findings provide substantial insight into the types of complications most often associated with litigation, the legal context in which these cases unfold, and the systemic vulnerabilities in current surgical practice that contribute to adverse medico-legal outcomes.

The overwhelming representation of studies from the USA underscores the highly litigious environment of American healthcare, where malpractice claims are systematically tracked and publicly recorded. This contrasts with countries that may experience underreporting due to limited access to legal representation, weaker regulatory oversight, or cultural barriers to litigation. Nevertheless, the presence of cases from Brazil, Canada, the UK, and Italy illustrates that litigation in aesthetic medicine is a global concern, with rising case volumes and increasing awareness among patients of their legal rights.

The majority of the reviewed studies employed retrospective methodologies, relying heavily on legal database searches (e.g. Westlaw, LexisNexis, CMPA) or national court archives. This methodological choice provides robust data for understanding past litigation but is limited in predicting future trends. Moreover, the retrospective nature makes it challenging to capture informal dispute resolutions and pre-trial settlements, which are common in aesthetic surgery malpractice claims. Future research could benefit from incorporating prospective registries and hospital-based litigation tracking systems [70–72].

Complications leading to legal claims were often preventable, including infection, wound dehiscence, and thromboembolic events [70–74]. The particularly high number of claims involving embolism, especially in liposuction and fat grafting procedures, signals a need for increased vigilance [75–78]. Despite improvements in surgical technique, these complications remain prevalent and are frequently lethal [75, 79–81]. Notably, a subset of fatal complications occurred in high-volume ambulatory centres, especially in regions with less stringent oversight, such as South Florida in the USA [82, 83]. This emphasises the need for regulatory reforms and uniform safety protocols for outpatient cosmetic surgery facilities.

The consistent theme across the studies was the central role of informed consent. More than one-third of claims were rooted in allegations of inadequate consent [84, 85]. The nature of aesthetic surgery, where subjective patient expectations play a significant role in satisfaction, elevates the importance of a comprehensive consent process [2, 23, 86, 87]. Surgeons who thoroughly documented the discussion of aesthetic procedure risks, benefits, alternatives, and limitations were more likely to avoid legal liability. This supports the implementation of standardised consent protocols, including visual tools and psychological screening for patients with unrealistic expectations or suspected body dysmorphic disorder (BDD) [5, 88–92].

The review also revealed procedural errors as another frequent allegation, often stemming from improper technique, unqualified operators (e.g. general practitioners performing aesthetic surgeries), or inadequate supervision in training settings. Cases involving non-specialists or surgeries performed outside their scope of certification had higher rates of adverse outcomes and successful patient claims [93, 94]. Legal literature supports restricting certain procedures to board-certified plastic surgeons and mandating continued education in patient safety [13, 75, 76].

Surprisingly, few studies addressed the role of postoperative follow-up in litigation risk [8, 70, 72, 95]. However, missed and poor communication during recovery were highlighted in qualitative reviews [23, 26, 28, 33]. This indicates that improving continuity of care and establishing reliable communication channels may reduce dissatisfaction and prevent legal escalation [96]. Additionally, in the context of medical tourism—another rising trend—patients treated abroad often lacked access to follow-up care, further compounding risks and legal ambiguity [16, 27, 97].

Financially, the data demonstrated that claims involving functional impairment (e.g. embolism, necrosis, chronic pain) resulted in higher compensation compared to claims driven purely by aesthetic dissatisfaction [27, 30, 43, 45, 54, 55, 59]. This suggests that courts give greater weight to objective injuries than to subjective appearance-related grievances, even in aesthetic surgery. Still, settlements were not uncommon even in subjective cases, particularly when documentation was vague or surgeons were unable to prove proper consent or protocol adherence.

The results presented in this study support previous findings in facial aesthetic surgery litigation, indicating that the underlying drivers of lawsuits, such as unmet expectations, inadequate, and poor communication, transcend anatomical regions [11, 16, 18, 27]. Body aesthetic surgeries may be uniquely vulnerable due to the scale and invasiveness of procedures such as BBL, abdominoplasty, or high-volume [27, 30, 43, 45, 47, 51, 54, 55, 59, 98]. These are also increasingly performed in outpatient settings, where emergency preparedness and monitoring may be compromised.

Additionally, findings indicate that litigation risk and patient dissatisfaction vary substantially by procedure type. Breast surgeries, especially cosmetic augmentation, were linked to subjective dissatisfaction and higher rates of informed consent disputes. In contrast, abdominoplasty patients often prioritised functional outcomes, and their legal claims focused more on wound complications than aesthetics. Gluteal procedures exhibited the most severe outcomes, with fat embolism being a leading cause of fatal litigation. This underscores the need for mandatory surgeon training for high-risk fat grafting. These insights support the development of procedure-specific consent protocols, risk stratification tolls, and postoperative safety checklist tailored to the complication of each surgery type.

## Conclusion

This systematic review highlights the complex interplay between aesthetic body surgery, patient outcomes, and medico-legal accountability. The findings reveal that inadequate informed consent, procedural errors, poor communication, and insufficient postoperative care remain the most common drivers of litigation.

Liposuction, abdominoplasty, gluteoplasty, and breast augmentation were the most litigated procedures, with complications such as infections, embolism, scarring, wound dehiscence, skin necrosis, haemorrhage/haematoma, and implant rupture frequently leading to legal claims. Gluteal fat grafting was associated with the highest mortality and legal risk. Across most countries studied, especially in the USA and Brazil, litigation was more likely to be successful for plaintiffs when documentation was lacking, or complications were not adequately communicated.

From a legal standpoint, the role of informed consent emerged as central. Surgeons who failed to document risk discussions or who operated on patients with unrealistic expectations faced a significantly higher probability of losing in court. In contrast, those who provided comprehensive preoperative counselling and maintained detailed records were generally better protected legally.

Psychological screening and expectation management should be emphasised in cosmetic breast and gluteal procedures, where subjective dissatisfaction is prevalent. Reconstructions, like abdominoplasty, benefit from documentation of medical indications and comorbidity management. Surgeons must apply differentiated risk communication strategies, ensuring patients understand the distinct risks and expected outcomes of each procedures type.

To mitigate these risks, several strategies are recommended: standardised, detailed informed consent protocols; adherence to procedural scope and guidelines; enhanced training in risk communication and medico-legal education; and incorporation of psychological screening for patients seeking body aesthetic procedures. Additionally, regulators should consider mandatory complication reporting systems and certification requirements for outpatient aesthetic practices. By implementing these strategies, the medical community can improve outcomes while minimising the legal and ethical risks inherent to aesthetic practice.

## Supplementary Information

Below is the link to the electronic supplementary material.

Supplementary file1 (DOCX 381 KB)
