# Supplementary material for: Litigation and Complications Arising from Aesthetic Body Surgery: A Systematic Review
Source: Aesthetic Plast Surg. 2025 Oct 15;50(1):241–58. doi: 10.1007/s00266-025-05276-y (PMC12916921; doi:10.1007/s00266-025-05276-y)
Supplement: Supplementary file 1 — Supplementary file1 (DOCX 381 KB) [file 266_2025_5276_MOESM1_ESM.docx]

*Supplementary Material*

**WHEN BEAUTY TURNS LITIGIOUS: WHAT DO WE KNOW ABOUT COMPLICATIONS AND LEGAL DISPUTES IN NON-SURGICAL FACIAL AESTHETICS****: A SYSTEMATIC REVIEW**

Table S1. PRISMA 2020 Checklist

| **Section and Topic** | **Item #** | **Checklist item** | **Location where item is reported** |
| --- | --- | --- | --- |
| **TITLE** | | |  |
| Title | 1 | Identify the report as a systematic review. | Title Page |
| **ABSTRACT** | | |  |
| Abstract | 2 | See the PRISMA 2020 for Abstracts checklist. | Abstract |
| **INTRODUCTION** | | |  |
| Rationale | 3 | Describe the rationale for the review in the context of existing knowledge. | Intro (pp. 1) |
| Objectives | 4 | Provide an explicit statement of the objective(s) or question(s) the review addresses. | Intro, final paragraph (p. 1) |
| **METHODS** | | |  |
| Eligibility criteria | 5 | Specify the inclusion and exclusion criteria for the review and how studies were grouped for the syntheses. | Methods: Eligibility (p. 2) |
| Information sources | 6 | Specify all databases, registers, websites, organisations, reference lists, and other sources searched or consulted to identify studies. Specify the date when each source was last searched or consulted. | Methods: Information sources (p. 2); Suppl. table S2-S4 |
| Search strategy | 7 | Present the full search strategies for all databases, registers and websites, including any filters and limits used. | Suppl. table S2-S4 |
| Selection process | 8 | Specify the methods used to decide whether a study met the inclusion criteria of the review, including how many reviewers screened each record and each report retrieved, whether they worked independently, and if applicable, details of automation tools used in the process. | Methods: Selection process (p. 3); Suppl. table S5; PRISMA flow diagram -Supplementary Material (eFig.S1). |
| Data collection process | 9 | Specify the methods used to collect data from reports, including how many reviewers collected data from each report, whether they worked independently, any processes for obtaining or confirming data from study investigators, and if applicable, details of automation tools used in the process. | Methods: Data collection and extraction (p. 3) |
| Data items | 10a | List and define all outcomes for which data were sought. Specify whether all results that were compatible with each outcome domain in each study were sought (e.g. for all measures, time points, analyses), and if not, the methods used to decide which results to collect. | Methods: p.1-4; Suppl. tables S7.1 and S7.2; Suppl. table S8; Results: Outcomes (pp. 4–8) |
|  | 10b | List and define all other variables for which data were sought (e.g. participant and intervention characteristics, funding sources). Describe any assumptions made about any missing or unclear information. | Methods: p.1-4; Suppl. tables S7.1 and S7.2 |
| Study risk of bias assessment | 11 | Specify the methods used to assess risk of bias in the included studies, including details of the tool(s) used, how many reviewers assessed each study and whether they worked independently, and if applicable, details of automation tools used in the process. | Methods: Risk of Bias Assessment (p. 3); |
| Effect measures | 12 | Specify for each outcome the effect measure(s) (e.g. risk ratio, mean difference) used in the synthesis or presentation of results. | Methods: Data Synthesis (p. 3); Results (p.4-8); ; Suppl. tables S12.1 and 12.2. |
| Synthesis methods | 13a | Describe the processes used to decide which studies were eligible for each synthesis (e.g. tabulating the study intervention characteristics and comparing against the planned groups for each synthesis (item #5)). | Methods: Data Synthesis (p. 3); Suppl. table S8 |
|  | 13b | Describe any methods required to prepare the data for presentation or synthesis, such as handling of missing summary statistics or data conversions. | Data Synthesis (p. 3); Suppl. tables S7 and S8 |
|  | 13c | Describe any methods used to tabulate or visually display results of individual studies and syntheses. | Methods: Data Synthesis (p. 3); Results: (pp. 4–8); Suppl. tables S7–S10; eFig. S1; Fig. 1 |
|  | 13d | Describe any methods used to synthesize results and provide a rationale for the choice(s). If meta-analysis was performed, describe the model(s), method(s) to identify the presence and extent of statistical heterogeneity, and software package(s) used. | Methods: Data Synthesis (p.3); Results: pp. 4–8; Suppl. tables S8; Appx Table S10 |
|  | 13e | Describe any methods used to explore possible causes of heterogeneity among study results (e.g. subgroup analysis, meta-regression). | Methods: Data Synthesis (p. 3); Results: pp. 4–8); Suppl. tables S7,8 |
|  | 13f | Describe any sensitivity analyses conducted to assess robustness of the synthesized results. | Methods: Data Synthesis (p. 3); Results: pp. 4-8. |
| Reporting bias assessment | 14 | Describe any methods used to assess risk of bias due to missing results in a synthesis (arising from reporting biases). | Methods: Certainty of Evidence (pp. 7,8); Discussion: Limitations (pp. 10,11); Suppl. Tables S10-S14 |
| Certainty assessment | 15 | Describe any methods used to assess certainty (or confidence) in the body of evidence for an outcome. | Certainty of Evidence (pp.7,8); Suppl. Tables S10-S14 |
| **RESULTS** | | |  |
| Study selection | 16a | Describe the results of the search and selection process, from the number of records identified in the search to the number of studies included in the review, ideally using a flow diagram. | Results: Study Selection (p.4); eFig. S1 (PRISMA 2020 Flow Diagram) |
|  | 16b | Cite studies that might appear to meet the inclusion criteria, but which were excluded, and explain why they were excluded. | Results: Study Selection (p. 4); Suppl. Table S5 |
| Study characteristics | 17 | Cite each included study and present its characteristics. | Results: Study Characteristics (pp. 4,5); Suppl. Tables S7 |
| Risk of bias in studies | 18 | Present assessments of risk of bias for each included study. | Results: Risk of Bias (pp. 12–13); Appx Tables S8–S10; eFig. S1 |
| Results of individual studies | 19 | For all outcomes, present, for each study: (a) summary statistics for each group (where appropriate) and (b) an effect estimate and its precision (e.g. confidence/credible interval), ideally using structured tables or plots. | Results: Study Characteristics and Outcomes (pp. 4,5); Suppl. Tables S6–S9 |
| Results of syntheses | 20a | For each synthesis, briefly summarise the characteristics and risk of bias among contributing studies. | Results: pp. 4–8; Suppl. Tables S6–S11 |
|  | 20b | Present results of all statistical syntheses conducted. If meta-analysis was done, present for each the summary estimate and its precision (e.g. confidence/credible interval) and measures of statistical heterogeneity. If comparing groups, describe the direction of the effect. | Results: pp. 4–8; Suppl. Tables S11-14 |
|  | 20c | Present results of all investigations of possible causes of heterogeneity among study results. | Results: pp. 4–8; Suppl. Tables S7,8 |
|  | 20d | Present results of all sensitivity analyses conducted to assess the robustness of the synthesized results. | Results: pp4-8; Suppl. Tables S10-14 |
| Reporting biases | 21 | Present assessments of risk of bias due to missing results (arising from reporting biases) for each synthesis assessed. | Results: Risk of Bias (pp. 6,7); Limitations (pp. 9,10); Suppl. Tables S8–S11; eFig. S1 |
| Certainty of evidence | 22 | Present assessments of certainty (or confidence) in the body of evidence for each outcome assessed. | Results: pp. 4-8; Suppl. Tables S6-14 |
| **DISCUSSION** | | |  |
| Discussion | 23a | Provide a general interpretation of the results in the context of other evidence. | Discussion (pp. 8-10) |
|  | 23b | Discuss any limitations of the evidence included in the review. | Discussion: Limitations (pp. 9,10) |
|  | 23c | Discuss any limitations of the review processes used. | Discussion: Limitations (pp. 9,10) |
|  | 23d | Discuss implications of the results for practice, policy, and future research. | Implications (p. 9) |
| **OTHER INFORMATION** | | |  |
| Registration and protocol | 24a | Provide registration information for the review, including register name and registration number, or state that the review was not registered. | ID: CRD420251134167 |
|  | 24b | Indicate where the review protocol can be accessed, or state that a protocol was not prepared. | PROSPERO |
|  | 24c | Describe and explain any amendments to information provided at registration or in the protocol. | p.1 |
| Support | 25 | Describe sources of financial or non-financial support for the review, and the role of the funders or sponsors in the review. | p.10 |
| Competing interests | 26 | Declare any competing interests of review authors. | p.10 |
| Availability of data, code and other materials | 27 | Report which of the following are publicly available and where they can be found: template data collection forms; data extracted from included studies; data used for all analyses; analytic code; any other materials used in the review. | Supplementary Material – e-format |

*From:*  Page MJ, McKenzie JE, Bossuyt PM, Boutron I, Hoffmann TC, Mulrow CD, et al. The PRISMA 2020 statement: an updated guideline for reporting systematic reviews. BMJ 2021;372:n71. doi: 10.1136/bmj.n71.

Table S2.**PICOS Search Strategy**

| ***Concept 1:*** | ***Concept 2:*** | ***Concept 3:*** | ***Concept 4:*** |
| --- | --- | --- | --- |
| Humans  OR  Facе | Aesthetic/esthetics procedure  OR  Non-surgical facial rejuvenation  OR  Minimally invasive aesthetic procedures  OR  Facial aesthetic treatments  OR  Non-invasive cosmetic techniques  OR  Plastic non-surgery procedures  OR  Esthetics  OR  Cosmetic procedure  OR  Aesthetic/esthetics treatment  OR  Non-surgical intervention  OR  Aesthetic/esthetics non-surgical interventions  OR  Fillers  OR  Dermal fillers  OR  Hyaluronic acid filler  OR  Hyaluronic acid  OR  Calcium hydroxylapatite  OR  Hydroxyapatite (HA)  OR  Polymethylmethacrylate  OR  Poly-L-lactic acid  OR  Botulinum toxin/botox  OR  Botulinum toxin type A  OR  Botulinum toxin type B  OR  Neuromodulators  OR  Facial muscle chemodenervation  OR  Autologous fat grafting  OR  Facial fat transfer  OR  Fat micrografting  OR  Thread-Based Lifting  OR  Thread lift  OR  Barbed sutures  OR  Polydioxanone threads  OR  Non-surgical facelifting  OR  Laser resurfacing  OR  Ablative laser  OR  Non-ablative laser  OR  Intense pulsed light (IPL)  OR  Laser hair removal  OR  Tattoo removal (laser)  OR  OR  Radiofrequency (RF) skin tightening  OR  High-intensity focused ultrasound (HIFU)  OR  Ultrasound-based facial rejuvenation  OR  Chemical peels  OR  Alpha hydroxy acids (AHAs)  OR  Beta hydroxy acids (BHAs)  OR  Microdermabrasion  OR  Mesotherapy  OR  Biostimulation  OR  Skin boosters  OR  Facial hair transplantation  OR  Beard transplantation  OR  Hairline restoration  OR  Follicular unit extraction (FUE) (Facial) | Forensic*  OR  Forensic medicine  OR  Medical-legal  OR  Malpractice  OR  Legislation  OR  Liability  OR  Legal liability  OR  Legal  OR  Compensation and redress  OR  Expert  OR  Litigation  OF  Insurance  OR  Negligence claims complaint  OR  Medical negligence  OR  Appearance quality  OR  Medicolegal implications  OR  Lawsuits /malpractice lawsuit | Dissatisfaction  OR  Cosmetic procedure victims  OR  Complications  OR  Adverse event  OR  Side effects  OR  Sequelae  OR  Medical malpractice  OR  Medical ethics  OR  Malpractice claims  OR  Medical practice error  OR  Medical error  OR  Patient safety  OR  Aesthetic outcomes  OR  Clinical efficacy  OR  Ethics in cosmetic medicine |

Table S3. Search Strategy for the Systematic Review

| **PICOS Database**  **Search Strategy** | *Search 1 (P):* Human* OR Face OR Facial  *Search 2 (I):* Aesthetic procedure OR Non-surgical rejuvenation OR Minimally invasive aesthetic procedures OR Non-invasive cosmetic techniques OR non-surgery procedures OR Esthetics OR Cosmetic procedure OR Fillers OR Hyaluronic acid OR Calcium hydroxylapatite OR Botulinum toxin/botoxOR Autologous fat grafting OR Thread lift OR Laser OR Chemical peels OR Skin boosters OR Facial hair transplantation  *Search 3 (C):*  *Search 4 (O):* (Forensic OR Medical-legal OR Malpractice OR Legal liability) AND (Dissatisfaction OR Complications OR Adverse event OR  Side effects OR Sequelae OR Medical malpractice)  ***Combined Searches***  Search 1 AND Search 2 AND Search 3 AND Search 4 |
| --- | --- |
| **Databases searched** | MEDLINE (Ovid)  EMBASE (Ovid)  Cochrane Library, including the Central Register of Controlled Trials (CENTRAL)  PubMed  Web of Science (Clarivate Analytics)  SCOPUS (Elsevier)  *Grey Literature Sources:*  WorldCat, ProQuest Dissertations & Theses (PQDT), Open Access Theses and Dissertations (OATD), SSRN, and F1000 Preprints. |
| **Part of the journals searched** | Keywords in the abstract and title  MeSH terms  Subject Heading Searching (Medline, Embase),  Subheadings (Medline, Embase ) |
| **Years of search** | from 1 January 2020 to 31 March 2025  Searches were updated to March 31, 2025 |
| **Language** | English language only |

Table S4. Search strategy adapted to each database

| **Electronic**  **Database** | **Search strategy used** | **Filter/**  **Results** |
| --- | --- | --- |
| **MEDLINE**  **via OVID** | **Ovid MEDLINE(R) ALL <1946 to April 14, 2025>**  1 exp Face/ 180303  2 (aesthetic* or esthetics* procedure).mp. or (exp Cosmetic Techniques/ or exp Dermal Fillers/) [mp=title, book title, abstract, original title, name of substance word, subject heading word, floating sub-heading word, keyword heading word, organism supplementary concept word, protocol supplementary concept word, rare disease supplementary concept word, unique identifier, synonyms, population supplementary concept word, anatomy supplementary concept word] 101553  3 exp Face/ or exp Cosmetic Techniques/ or exp Humans/ or cosmetic procedure.mp. or exp Botulinum Toxins, Type A/ 22684700  4 exp Face/ or exp Humans/ or exp Esthetics/ or exp Dermal Fillers/ or facial aesthetic procedure.mp. or exp Cosmetic Techniques/ or exp Skin Aging/ 22684374  5 (aesthetic* or esthetics* treatment).mp. [mp=title, book title, abstract, original title, name of substance word, subject heading word, floating sub-heading word, keyword heading word, organism supplementary concept word, protocol supplementary concept word, rare disease supplementary concept word, unique identifier, synonyms, population supplementary concept word, anatomy supplementary concept word] 46303  6 exp Face/ or exp Humans/ or exp Botulinum Toxins, Type A/ or Facial non-surgical interventions.mp. 22682983  7 Facial* non-surgical interventions.mp. 0  8 (Face aesthetic* or esthetics* non-surgical interventions).mp. [mp=title, book title, abstract, original title, name of substance word, subject heading word, floating sub-heading word, keyword heading word, organism supplementary concept word, protocol supplementary concept word, rare disease supplementary concept word, unique identifier, synonyms, population supplementary concept word, anatomy supplementary concept word] 46  9 exp Dermal Fillers/ or exp Cosmetic Techniques/ or Fillers.mp. or exp Hyaluronic Acid/ or exp Biocompatible Materials/ 240838  10 exp Hyaluronic Acid/ or exp Cosmetic Techniques/ or exp Dermal Fillers/ or Face* fillers.mp. or exp Humans/ or exp Face/ 22693132  11 (botulinum toxin or botox).mp. [mp=title, book title, abstract, original title, name of substance word, subject heading word, floating sub-heading word, keyword heading word, organism supplementary concept word, protocol supplementary concept word, rare disease supplementary concept word, unique identifier, synonyms, population supplementary concept word, anatomy supplementary concept word] 19586  12 hyaluronic acid.mp. or exp Hyaluronic Acid/ 41034  13 (hydroxyapatite or HA).mp. [mp=title, book title, abstract, original title, name of substance word, subject heading word, floating sub-heading word, keyword heading word, organism supplementary concept word, protocol supplementary concept word, rare disease supplementary concept word, unique identifier, synonyms, population supplementary concept word, anatomy supplementary concept word] 136612  14 1 or 2 or 3 or 4 or 5 or 6 or 7 or 8 or 9 or 10 or 11 or 12 or 13 22856156  15 exp Forensic Medicine/ or Forensic*.mp. 150069  16 exp Forensic Medicine/ or forensic medicine.mp. 112109  17 medical-legal.mp. 1902  18 malpractice.mp. or exp Malpractice/ 36073  19 legislation.mp. or exp Legislation/ or exp Legislation, Medical/ 333394  20 exp Liability, Legal/ or exp Insurance, Liability/ or liability.mp. 39388  21 legal.mp. or exp Liability, Legal/ 140461  22 (compensation and redress).mp. [mp=title, book title, abstract, original title, name of substance word, subject heading word, floating sub-heading word, keyword heading word, organism supplementary concept word, protocol supplementary concept word, rare disease supplementary concept word, unique identifier, synonyms, population supplementary concept word, anatomy supplementary concept word] 3244  23 exp Expert Testimony/ or Expert.mp. 166117  24 litigation.mp. or exp Jurisprudence/ 224732  25 exp Insurance, Liability/ 6023  26 exp Medical Errors/ or exp Malpractice/ or Negligence Claims complaint.mp. 155222  27 exp Diagnostic Errors/ or exp Medical Errors/ or exp Malpractice/ or medical negligence.mp. or exp Risk Management/ or exp Liability, Legal/ or exp Informed Consent/ 595881  28 appearance quality.mp. 282  29 medicolegal* implications.mp. 494  30 (Lawsuits or malpractice lawsuit).mp. [mp=title, book title, abstract, original title, name of substance word, subject heading word, floating sub-heading word, keyword heading word, organism supplementary concept word, protocol supplementary concept word, rare disease supplementary concept word, unique identifier, synonyms, population supplementary concept word, anatomy supplementary concept word] 2455  31 15 or 16 or 17 or 18 or 19 or 20 or 21 or 22 or 23 or 24 or 25 or 26 or 27 or 28 or 29 or 30 1309669  32 14 and 31 1091554  33 1 or 2 or 7 268550  34 31 and 33 8868  35 15 or 16 or 17 or 18 or 19 or 20 509724  36 33 and 35 3126  37 2 and 18 and 19 and 20 26 | **Language:** no filter  **Publication date:** 2020  onwards  **Hits: 26** |
| **Embase**  **via OVID** | Embase Classic+Embase <1947 to 2025 April 14>  1 exp esthetics/ or exp botulinum toxin A/ or exp face/ or exp cosmetic/ or non-surgical facial cosmetic procedures.mp. 343180  2 (Fillers or face fillers).mp. [mp=title, abstract, heading word, drug trade name, original title, device manufacturer, drug manufacturer, device trade name, keyword heading word, floating subheading word, candidate term word] 10086  3 exp filler/ or Fillers.mp. or exp hyaluronic acid/ 286832  4 exp human/ or exp hyaluronic acid/ or exp face/ or face fillers.mp. or exp filler/ or exp botulinum toxin A/ 29328979  5 1 or 2 or 3 or 4 29361453  6 malpractice.mp. or exp malpractice/ 39036  7 (Forensic* or forensic medicine or medical-legal).mp. [mp=title, abstract, heading word, drug trade name, original title, device manufacturer, drug manufacturer, device trade name, keyword heading word, floating subheading word, candidate term word] 128983  8 legislation.mp. or exp law/ 185482  9 exp malpractice/ or exp legal liability/ or exp medical liability/ or liability.mp. or exp legal aspect/ 1487096  10 litigation.mp. or exp law suit/ 19104  11 6 or 7 or 8 or 9 or 10 1605081  12 5 and 11 1262732  13 4 and 6 29217  14 4 and 9 1176692  15 1 or 4 29358403  16 9 and 15 1177415  17 6 and 15 29219  18 17 and 2020:2025.(sa_year). 2728  19 4 and 9 1176692  20 (exp human/ or exp hyaluronic acid/ or exp face/ or face fillers.mp. or exp filler/ or exp botulinum toxin A/) and non-surgical facial cosmetic procedures.mp. 1  21 exp human/ or exp hyaluronic acid/ or exp face/ or face fillers.mp. or exp filler/ or exp botulinum toxin A/ 29328979  22 ((((malpractice.mp. or exp malpractice/) and litigation.mp.) or exp law suit/) and legislation.mp.) or exp law/ 132631  23 21 and 22 77288  24 face.mp. or exp face/ 603744  25 exp botulinum toxin A/ or cosmetic procedures.mp. or exp laser/ or exp cosmetic/ 377975  26 ((((forensic* and malpractice) or legislation or liability) and legal) or compensation).mp. [mp=title, abstract, heading word, drug trade name, original title, device manufacturer, drug manufacturer, device trade name, keyword heading word, floating subheading word, candidate term word] 130615  27 24 and 25 16007  28 26 and 27 21  29 face non-surgical cosmetic procedures.mp. 0  30 FACE.mp. or exp face/ 603744  31 exp botulinum toxin/ or exp cosmetic/ or exp hyaluronic acid/ or non-surgical cosmetic procedures.mp. 181503  32 non-surgical cosmetic procedures.mp. 23  33 30 and 31 and 32 3  34 26 and 33 0  35 court.mp. or exp court/ 36944  36 30 and 35 712  37 30 and 31 and 35 4  38 31 and 35 55  39 30 and 31 and 35 4  40 38 and 2020:2025.(sa_year). 15  41 4 and 22 and 35 4137  42 41 and 2020:2025.(sa_year). 705  43 4 and 6 and 35 1660  44 43 and 2020:2025.(sa_year). 253  45 1 and 35 120  46 45 and 2020:2025.(sa_year). 31  *Searches were updated to March 31, 2025* | **Language:** no filter  **Publication date:** 2020 onwards  **Hits:31** |
| **Cochrane Library, including Central Register of Controlled Trials (CENTRAL)** | Date Run: 31/03/2025 11:38:51  ID Search Hits  #2 "genital microbiome" OR "genital microorganisms" OR "genitals micro" OR "genital bacterial communities" OR "genital tract microbiota" OR "bacterial flora of the genitourinary tract" OR "microbial consortium male and female" OR "genital microbial genomic" OR "urogenital microbiome" 16  #3 ("individual identification of genital microbiome" OR "microbiome personal identity" OR "microbiomes identification" OR "microbiome human identification" OR "changes microbiota after sex" OR "Complementary seminovaginal microbiome" OR "varied microbiota after sex" OR "sexual intercourse" OR "genital contact") 2491  #4 ("forensic examination" OR "forensic medicine" OR "evidence of sexual assault" OR "evidence of rape") 490  #5 Sex OR Sexual Or sexually OR Sexual assault OR sexual intercourse 95682  #6 #2 OR #3 AND #4 AND #5 with Cochrane Library publication date Between Jan 2020 and Jun 2025 12  #7 Humans OR adults 1004080  #8 (face fillers OR botulinum toxin OR botox OR hyaluronic acid OR facial aesthetic OR hydroxyapatite OR cosmetic procedure OR aesthetic* treatment OR esthetics* procedure OR Non-surgical aesthetic* interventions):ti,ab,kw NOT (surgical):ti,ab,kw (Word variations have been searched) with Publication Year from 2020 to 2025, in Trials 4621  #9 (litigation OR forensic OR legal):ti,ab,kw AND ("court trial"):ti,ab,kw (Word variations have been searched) 2  #10 (litigation OR forensic OR legal):ti,ab,kw (Word variations have been searched) 14029  #11 #8 AND #10 AND # 14 3  #12 #8 AND #10 with Publication Year from 2020 to 2025, in Trials 29  #13 Humans OR adults 1004080  #14 (face fillers OR botulinum toxin OR botox OR hyaluronic acid OR facial aesthetic OR hydroxyapatite OR cosmetic procedure OR aesthetic* treatment OR esthetics* procedure OR Non-surgical aesthetic* interventions):ti,ab,kw NOT (surgical):ti,ab,kw (Word variations have been searched) with Publication Year from 2020 to 2025, in Trials 4621  #15 (litigation OR forensic OR legal):ti,ab,kw AND ("court trial"):ti,ab,kw (Word variations have been searched) 2  #16 (litigation OR forensic OR legal):ti,ab,kw (Word variations have been searched) 14029  #17 #14 AND #16 AND # 14 3  #18 #14 AND #16 with Publication Year from 2020 to 2025, in Trials 29  *Searches were updated to March 31, 2025* | **Language:** no filter  **Publication date:** 2020 onwards  **Hits: 29** |
| **PubMed** | (face fillers OR botulinum toxin OR botox OR hyaluronic acid OR facial aesthetic OR hydroxyapatite OR cosmetic procedure OR aesthetic procedure) AND malpractice -39  *Searches were updated to March 31, 2025* | **Language:** no filter  **Publication date:** from 2020 onwards  **Hits: 39** |
| **Web of Science**  Core Collection  *(Clarivate Analytics****)*** | Date Run: Feb 02 2025  face fillers OR botulinum toxin OR botox OR hyaluronic acid OR facial aesthetic procedure OR hydroxyapatite OR cosmetic procedure OR esthetics procedure OR Non-surgical interventions OR laser* OR peeling* (All Fields) and court OR medico-legal OR forens* OR forensic medicine OR malpractice OR legal OR liability OR legislation OR Medical malpractice OR medical error* OR Malpractice claims OR Medical error (All Fields) and dissatisfaction cosmetic procedure OR victims cosmetic procedure OR Non-surgical interventions problem OR dissatisfaction OR facial* aesthetic procedure (All Fields)  Timespan: 2020-01-01 to 2025-04-01 (Publication Date)  *Searches were updated to March 31, 2025*  *[https://www.webofscience.com/wos/woscc/summary/156d083e-5ce3-4e02-800d-e9847a5f5de3-015b983f2a/relevance/1 43](https://www.webofscience.com/wos/woscc/summary/156d083e-5ce3-4e02-800d-e9847a5f5de3-015b983f2a/relevance/1%2043)* | **Language:** no filter  **Publication date:** from 2020 onwards  **Hits: 43** |
| **Scopus**  **(Elsevier)** | ( TITLE-ABS-KEY ( "face* fillers" OR "botulinum toxin" OR botox OR "hyaluronic acid" OR "facial aesthetic procedure" OR hydroxyapatite OR "aesthetic* cosmetic procedure" OR "esthetics procedure" OR laser OR peeling ) AND TITLE-ABS-KEY ( court OR malpractice OR forensic* OR medical-legal OR litigation AND malpractice OR insurance OR "Negligence Claims complaint" OR "appearance quality" OR "lawsuit" ) ) AND PUBYEAR > 2019 AND PUBYEAR < 2026  *Searches were updated to March 31, 2025* | **Language:** no filter  **Publication date:** from 2020 onwards  **Hits:42** |
| **Citation searching** | **The literature search was supplemented by examining references in the relevant articles found. We used the Science Citation Index and Social Sciences Citation Index in Web of Science to track relevant citations.** | **Language:** no filter  **Publication date:** from 2020 onwards  **Hits: 107** |
| **ClinicalTrials.gov** | **<https://clinicaltrials.gov/>**  “Fillers” OR “laser” OR “facial cosmetic/aesthetic non-surgical procedure"  Searched 31 May 2025 (1 record) | No filter |
| **WHO ICTRP** | **<https://trialsearch.who.int/>**  Searched February 2025 (1 record) | No filter |
| ***Grey literature*** | | |
| **WorldCat** | <https://search.worldcat.org/topics/welcome>  Searched *March 31, 2025* (4 records)  ti: Face aesthetic procedure (article)  Timespan: 2020 to 2025 | **Hits: 4** |
| **ProQuest Dissertations**  **& Theses**  **(PQDT)** | <https://proquest.libguides.com/pqdt>  "aesthetic procedure"  Timespan: 2020 to 2025  Searches were updated to June 15, 2025 | **Hits: 33** |
| **Open Access Theses and Dissertations**  **(OATD)** | <https://oatd.org/>  Searched 31 May 2025 (4 records)  (Face) AND (Fillers OR Lasers) AND (complications) NOT (Surgical)  Searches were updated to 31 May 2025 | **Hits: 4** |
| **SSRN** | <https://www.ssrn.com/index.cfm/en/>  Searched June 14, 2025  "aesthetic procedure"  Searches were updated to June 15, 2025 | **Hits: 8** |
| **F1000** | <https://f1000research.com/>  “Minimally invasive aesthetic face procedures complications”  Searches were updated to 31 May 2025 | **Hits: 3** |

Table S5. Excluded studies and reasons

| **S/N** | **Study** | **Reason** |
| --- | --- | --- |
|  | Alsaidan M, Abuyassin A, Alammar H, Hussein G. Prevalence and Quality of Informed Consent for Patients Undergoing Cosmetic Procedures: A Cross Sectional Study. Acta bioethica. 2021;27:37-48. doi: 10.4067/S1726-569X2021000100037. | The study does not involve consequences of malpractice or complications of non-surgical aesthetic procedures.  *No Medico-Legal or Complication Data (Aesthetic Efficacy Only)* |
|  | Ascher B, Rzany B, Kestemont P, Hilton S, Heckmann M, Bodokh I, Noah EM, Boineau D, Kerscher M, Volteau M, Le Berre P, Picaut P. Significantly Increased Patient Satisfaction Following Liquid Formulation A bobotulinumtoxin A Treatment in Glabellar Lines: FACE-Q Outcomes From a Phase 3 Clinical Trial. Aesthet Surg J. 2020;40(9):1000-1008. doi: 10.1093/asj/sjz248. | The study does not involve consequences of malpractice or complications of non-surgical aesthetic procedures.  *No Medico-Legal or Complication Data (Aesthetic Efficacy Only)* |
|  | Carneiro D, Fernandes G, Musse J, Marques J. Hyaluronic acid lip augmentation and its effect on human identification by cheiloscopy: a case report. Revista Brasileira de Odontologia Legal. 2024. doi: 10. 10.21117/rbol-v10n32023-511. | The study does not involve consequences of malpractice or complications of non-surgical aesthetic procedures.  *No Medico-Legal or Complication Data (Aesthetic Efficacy Only)* |
|  | Eftekhari MH, Aghaei H, Kangari H, Bahrami M, Eftekhari S, Tabatabaee SM, Shahraki K, Bahrami M, Broumand MG. Abobotulinum toxin A for periorbital facial rejuvenation: impact on ocular refractive parameters. Clin Exp Optom. 2021;104(1):115-118. doi: 10.1111/cxo.13117. | The study does not involve consequences of malpractice or complications of non-surgical aesthetic procedures.  *No Medico-Legal or Complication Data (Aesthetic Efficacy Only)* |
|  | Braz A, Colucci L, Macedo de Oliveira L, Monteiro G, Ormiga P, Wanick F, Cazerta C, Kerson G, Musumeci M, Silberberg M. A Retrospective Analysis of Safety in Participants Treated with a Hybrid Hyaluronic Acid and Calcium Hydroxyapatite Filler. Plast Reconstr Surg Glob Open. 2024;12(2):e5622. doi: 10.1097/GOX.0000000000005622. | The study does not involve consequences of malpractice or complications of non-surgical aesthetic procedures.  *No Medico-Legal or Complication Data (Aesthetic Efficacy Only)* |
|  | Ghanian S, Wambier SPF, Wambier CG. Mitigation of risk of botulinum toxin A-induced blepharoptosis with hyperconcentrated glabellar injections. J Am Acad Dermatol. 2024;90(6):1309-1310. doi: 10.1016/j.jaad.2023.12.025. | The study does not involve consequences of malpractice or complications of non-surgical aesthetic procedures.  *No Medico-Legal or Complication Data (Aesthetic Efficacy Only)* |
|  | Ahmed MB, Almohannadi FS, Shraim BA, Aljassem G, Al-Lahham S, Alsherawi A. Surgical Written Consent in Aesthetic Plastic Surgery: A Plastic Center Audit of Surgical Consent Standards. Cureus. 2024;16(1):e51701. doi: 10.7759/cureus.51701. | Study linked to surgical malpractice, not non-surgical cosmetic aesthetic procedures  *No Medico-Legal or Complication Data (Aesthetic Efficacy Only)* |
|  | Hussein A, Salman F. Patient Perception and Preferences in Choosing a Healthcare Provider for Non-Surgical Facial Aesthetic Procedures. Acta Scientific Medical Sciences. 2024;8. doi: 10.31080/ASMS.2024.08.1909. | The study is not related to non-surgical cosmetic aesthetic procedures of the face.  *No Medico-Legal or Complication Data (Aesthetic Efficacy Only)* |
|  | Idjianto L, Suyatna I, Astuti I. Legal Protections of Pratama Clinic Doctors in Providing Aesthetic Services. Journal of Law, Politics and Humanities. 2025;5:2393-2399. doi: 10.38035/jlph.v5i4.1465. | The study is not related to non-surgical cosmetic aesthetic procedures of the face.  *No Medico-Legal or Complication Data (Aesthetic Efficacy Only)* |
|  | Nikolis A, Cohen JL, Enright KM, Avelar L, Beleznay K, Biesman B, Cartier H, Cotofana S, Fabi S, Fitzgerald R, Goodman G, Lee W, Parada M, Rzany B, Schelke L, Wang H, Bromée T, Weiner S. Deliberations of the Safety Task Force: Risk factors and treatment of adverse events associated with aesthetic injectables. J Cosmet Dermatol. 2024;23(11):3551-3564. doi: 10.1111/jocd.16476. | The study does not involve consequences of malpractice or complications of non-surgical aesthetic procedures.  *Irrelevant or Non-Specific Focus* |
|  | Cavallini M, Casasco A, Ferrara F, Raichi M, Spinelli G. Hyaluronic Acid Fillers, Needle Contamination by Fastidious Microorganisms, and Risk of Complications. Dermatol Surg. 2023;49(2):161-163. doi: 10.1097/DSS.0000000000003674. | The study does not involve consequences of malpractice or complications of non-surgical aesthetic procedures.  *Irrelevant or Non-Specific Focus* |
|  | Hanganu B, Iorga M, Pop LM, Ioan BG. Socio-Demographic, Professional and Institutional Characteristics That Make Romanian Doctors More Prone to Malpractice Complaints. Medicina (Kaunas). 2022;58(2):287. doi: 10.3390/medicina58020287. | The study is not related to non-surgical cosmetic aesthetic procedures of the face.  *Irrelevant or Non-Specific Focus* |
|  | Lehmann L, Appelbaum S, Ostermann T, Weber B, Hofmann SC. Medical error analysis in dermatology according to the reports of the North Rhine Medical Association from 2004 to 2018. J Dtsch Dermatol Ges. 2022;20(12):1603-1611. doi: 10.1111/ddg.14899. | The study presents a broad analysis of medical errors in dermatology, without specific focus on non-surgical facial aesthetic procedures or their medico-legal consequences. It lacks targeted data on the legal or malpractice implications related to aesthetic interventions.  *Irrelevant or Non-Specific Focus* |
|  | Liang F, Liu J, Zhou H, Liu P. Inequality in the last resort: how medical appraisal affects malpractice litigations in China. Int J Legal Med. 2021;135(3):1047-1054. doi: 10.1007/s00414-020-02386-x. | The study is not related to non-surgical cosmetic aesthetic procedures of the face.  *Irrelevant or Non-Specific Focus* |
|  | Wang JV, Albornoz CA, Goldbach H, Mesinkovska N, Rohrer T, Zachary CB, Saedi N. Experiences With Medical Spas and Associated Complications: A Survey of Aesthetic Practitioners. Dermatol Surg. 2020;46(12):1543-1548. doi: 10.1097/DSS.0000000000002344. | It does not contain primary or secondary medico-legal data, nor does it directly assess malpractice or legal consequences of specific non-surgical facial cosmetic procedures.  *Irrelevant or Non-Specific Focus* |
|  | Lim YH, Saberi SA, Kamal K, Jalian HR, Avram M. Retrospective Analysis of US Litigations Involving Dermatologists From 2011 to 2022. Dermatol Surg. 2024;50(6):518-522. doi: 10.1097/DSS.0000000000004142. | The study is not related to non-surgical cosmetic aesthetic procedures of the face.  *Irrelevant or Non-Specific Focus* |
|  | Marion T, Werbel T, Torres A. Reducing Legal Risks and Social Media Issues for Cosmetic Surgery. Facial Plast Surg Clin North Am. 2023;31(2):333-340. doi: 10.1016/j.fsc.2023.01.017. | The study is not related to non-surgical cosmetic aesthetic procedures of the face.  *Irrelevant or Non-Specific Focus* |
|  | Patel AA, Garg SP, Varghese J, Alleyne B, Williams T, Huffman K, Ellis M, Galiano RD. A Comparative Analysis of Online Reporting of Possible Complications for Minimally Invasive Cosmetic Procedures. Eplasty. 2023;23:e17. PMID: 37187869. | The study is not related to non-surgical cosmetic aesthetic procedures of the face.  *Irrelevant or Non-Specific Focus* |
|  | Qin J, Lu B, Li X, Sun D, Liu R, Wu Y, Fan G. Integrating Complaint Analysis into Hospital Management: A Comparative Study of Surgical and Non-Surgical Complaints. Br J Hosp Med (Lond). 2024;85(8):1-17. doi: 10.12968/hmed.2024.0219. | The study is not related to non-surgical cosmetic aesthetic procedures of the face.  *Irrelevant or Non-Specific Focus* |
|  | Muttaqin M, Amelia R. Review of Vulnerability Theory on Patients' Rights in Medical Malpractice Cases. International Journal of Law Dynamics Review. 2024:2;111-120. doi: 10.62039/ijldr.v2i2.52. | The study is not related to non-surgical cosmetic aesthetic procedures of the face.  *Irrelevant or Non-Specific Focus* |
|  | Nittari G, Savva D, Gibelli F, Vulcanescu D, De Leo D, Ricci G. Anatomy, Etiology, Management, and Medico-Legal Implications of Botulinum-induced Blepharoptosis. Curr Rev Clin Exp Pharmacol. 2025;20(1):32-37. doi: 10.2174/0127724328310459240809073519. | The study is not related to non-surgical cosmetic aesthetic procedures of the face.  *Irrelevant or Non-Specific Focus* |
|  | Taniguchi K, Watari T, Nagoshi K. Characteristics and trends of medical malpractice claims in Japan between 2006 and 2021. PLoS One. 2023;18(12):e0296155. doi: 10.1371/journal.pone.0296155. | The study is not related to non-surgical cosmetic aesthetic procedures of the face.  *Irrelevant or Non-Specific Focus* |
|  | Vural T, Erbaş M, Balcı Y, Baysal İ. Evaluatıon of forensic medical aspects of medical interventions and damages caused by unauthorized persons. Leg Med (Tokyo). 2024;69:102450. doi: 10.1016/j.legalmed.2024.102450. | The study is not related to non-surgical cosmetic aesthetic procedures of the face.  *Irrelevant or Non-Specific Focus* |
|  | Warde MJ, Nunes MPT, Yarshell FL. Legal therapeutic forms for litigation between doctors and patients. Rev. Bras. Cir. Plást. 2022;37:388-398. [doi:10.5935/2177-1235.2022RBCP.553-en](http://www.dx.doi.org/10.5935/2177-1235.2022RBCP.553-en). | The study is not related to non-surgical cosmetic aesthetic procedures of the face.  *Irrelevant or Non-Specific Focus* |
|  | Rumancik B, Keele BJ, Rahnama-Moghadam S. Characterization of medical malpractice lawsuits relating to dermatologic emergencies in the inpatient and emergency settings. J Am Acad Dermatol. 2022;86(2):445-446. doi: 10.1016/j.jaad.2020.02.015. | The study is not related to non-surgical cosmetic aesthetic procedures of the face.  *Irrelevant or Non-Specific Focus* |
|  | Chayangsu O, Wanitphakdeedecha R, Pattanaprichakul P, Hidajat IJ, Evangelista KER, Manuskiatti W. Legal vs. illegal injectable fillers: The adverse effects comparison study, J. Cosmet. Dermatol. 2020;19:1580-1586. doi:10.1111/jocd.13492. | The study is not related to legal medicine and complications from non-surgical aesthetic procedures.  *Irrelevant or Non-Specific Focus* |
|  | Tran AQ, Lee WW. Vision Loss and Blindness Following Fillers. J Dermatol Skin Sci. 2021;3(2):1-4. PMID: 34296217. | *Review Articles Without Primary/Secondary Legal or Clinical Data* |
|  | Atiyeh BS, Chahine F, Abou Ghanem O. Social Media and Plastic Surgery Practice Building: A Thin Line Between Efficient Marketing, Professionalism, and Ethics. Aesthetic Plast Surg. 2021;45(3):1310-1321. doi: 10.1007/s00266-020-01961-2. | *Review Articles Without Primary/Secondary Legal or Clinical Data* |
|  | Kyriazidis I, Spyropoulou GA, Zambacos G, Tagka A, Rakhorst HA, Gasteratos K, Berner JE, Mandrekas A. Adverse Events Associated with Hyaluronic Acid Filler Injection for Non-surgical Facial Aesthetics: A Systematic Review of High Level of Evidence Studies. Aesthetic Plast Surg. 2024;48(4):719-741. doi: 10.1007/s00266-023-03465-1. | *Review Articles Without Primary/Secondary Legal or Clinical Data* |
|  | Foster J, Aakalu VK, Freitag SK, McCulley TJ, Tao JP, Vagefi MR, Yen MT, Kim SJ, Wladis EJ. Vision-Threatening Complications of Soft Tissue Fillers: A Report by the American Academy of Ophthalmology, Ophthalmology. 2025. doi:10.1016/j.ophtha.2025.01.020. | *Review Articles Without Primary/Secondary Legal or Clinical Data* |
|  | Deng K, Deng X, Luo H, Chen L, Liu Y, Wang J, Huang M, Hu J, Li T, Zhou J. Academic visualization study of aesthetic medicine management and related legal research since 2000. J Cosmet Dermatol. 2024;23(8):2697-2710. doi: 10.1111/jocd.16327. | *Review Articles Without Primary/Secondary Legal or Clinical Data* |
|  | Hibler BP, Eliades PJ, Kagha KC, Avram MM. Litigation Arising From Minimally Invasive Cosmetic Procedures: A Review of the Literature. Dermatol Surg. 2021;47(12):1606-1613. doi: 10.1097/DSS.0000000000003202. | *Review Articles Without Primary/Secondary Legal or Clinical Data* |
|  | Mehta N, Sharma A, Sindhuja T, Dudani P, Sahni K, Khanna N, Gupta S. Procedural dermatology and its unmet need. Indian J Dermatol Venereol Leprol. 2023;89(6):807-818. doi: 10.25259/IJDVL_322_2022. | *Review Articles Without Primary/Secondary Legal or Clinical Data* |
|  | Zein M, Tie-Shue R, Pirakitikulr N, Lee WW. Complications after cosmetic periocular filler: prevention and management. Plast Aesthet Res. 2020;7:44. doi: 10.20517/2347-9264.2020.133. | *Review Articles Without Primary/Secondary Legal or Clinical Data* |
|  | Madan R, Das N, Patley R, Nagpal N, Malik Y, Math SB. Consequences of medical negligence and litigations on health care providers - A narrative review. Indian J Psychiatry. 2024;66(4):317-325. doi: 10.4103/indianjpsychiatry.indianjpsychiatry_799_23. | *Review Articles Without Primary/Secondary Legal or Clinical Data* |
|  | Patel J, Otto E, Taylor JS, Mostow EN, Vidimos A, Lucas J, Khetarpal S, Regotti K, Kaw U. Patient safety in dermatology: a ten-year update. Dermatol Online J. 2021;27(3):13030/qt9cp0t2wt. PMID: 33865273. | *Review Articles Without Primary/Secondary Legal or Clinical Data* |
|  | Rahman E, Philipp-Dormston WG, Webb WR, Rao P, Sayed K, Sharif AQMO, Yu N, Ioannidis S, Tam E, Rahman Z, Mosahebi A, Goodman GJ. "Filler-Associated Acute Stroke Syndrome": Classification, Predictive Modelling of Hyaluronidase Efficacy, and Updated Case Review on Neurological and Visual Complications. Aesthetic Plast Surg. 2024;48(17):3222-3253. doi: 10.1007/s00266-024-04202-y. | *Review Articles Without Primary/Secondary Legal or Clinical Data* |
|  | Wollina U, Goldman A. Filler Migration after Facial Injection-A Narrative Review. Cosmetics. 2023;10:115. doi: 10.3390/cosmetics10040115. | *Review Articles Without Primary/Secondary Legal or Clinical Data* |
|  | Samizadeh S, De Boulle K. Complications of toxins and fillers in facial aesthetics. Prim Dent J. 2023;12(3):65-72. doi: 10.1177/20501684231197717. | *Review Articles Without Primary/Secondary Legal or Clinical Data* |
|  | Beutler K, Rzepczyk S, Bijata B, Lewandowski J, Świderski P. Complications in aesthetic medicine resulting from procedures that use fractional lasers in skin regeneration treatments, Aesthetic Medicine. 2024:10 doi:10.57662/am.v10i3.15719. | *Review Articles Without Primary/Secondary Legal or Clinical Data* |
|  | Cataneo JL, Mathis SA, Bartelt K, Gelfond A, Arias-Serrato R, Patel PA. Developing the Aesthetic Postoperative Complication Score (APeCS) for Detecting Major Morbidity in Facial Aesthetic Surgery. Aesthet Surg J. 2024;44(5):463-469. doi: 10.1093/asj/sjad379. | The study does not involve consequences of malpractice or complications of non-surgical aesthetic procedures.  *Surgical or Mixed Surgical Procedures* |
|  | Choinski K, Sanon O, Sacknovitz Y, Ilonzo N, Ting W, Koleilat I, Phair J. Review of Malpractice Litigation in the Diagnosis and Treatment of Venous & Lymphatic Disease. Ann Vasc Surg. 2023;88:274-282. doi: 10.1016/j.avsg.2022.07.002. | The study is not related to medical malpractice or complications of non-surgical facial aesthetic procedures  *Surgical or Mixed Surgical Procedures* |
|  | Boyke AE, Naidu I, Lam S, Alvi MA, Bader ER, Agarwal V. Medical Malpractice and Trigeminal Neuralgia: An Analysis of 49 Cases. J Oral Maxillofac Surg. 2021;79(5):1026.e1-1026.e8. doi: 10.1016/j.joms.2020.12.041. | The study is related to the negligence of surgeons and dentists, not to the performance of cosmetic aesthetic procedures.  *Surgical or Mixed Surgical Procedures* |
|  | Cotroneo DA, Russo I, Pallante A, Di Mauro L, Chisari M. Can appearance be deceiving? A Strange Case of Nasal AVM. Clin Ter. 2025;176(Suppl 1(2)):14-18. doi: 10.7417/CT.2025.5179. | Study linked to surgical malpractice, not non-surgical cosmetic aesthetic procedures  *Surgical or Mixed Surgical Procedures* |
|  | Feola A, Minotti C, Marchetti D, Caricato M, Capolupo GT, Marsella LT, La Monaca G. A Five-Year Survey for Plastic Surgery Malpractice Claims in Rome, Italy. Medicina (Kaunas). 2021;57(6):571. doi: 10.3390/medicina57060571. | Study linked to surgical malpractice, not non-surgical cosmetic aesthetic procedures  *Surgical or Mixed Surgical Procedures* |
|  | Halepas S, Muchemi F, Higham ZL, Ferneini EM. The Past Decade in Courts, What Oral-Maxillofacial Surgery Should Know About Facial Cosmetic Surgery. J Oral Maxillofac Surg. 2021;79(8):1743-1749. doi: 10.1016/j.joms.2021.04.007. | Study linked to surgical malpractice, not non-surgical cosmetic aesthetic procedures  *Surgical or Mixed Surgical Procedures* |
|  | Hamdan M, Ramli MA, Hehsan A, Nur AR, Muhammad MS, Syamsul M. The Application of Maqasid-Oriented Approach in Islamic Bioethics: A Case Study on Fatwa Related to Cosmetic, Plastic and Reconstructive Surgery. IIUM Medical Journal Malaysia. 2020:20. doi:10.31436/imjm.v20i1.1781. | Study linked to surgical malpractice, not non-surgical cosmetic aesthetic procedures  *Surgical or Mixed Surgical Procedures* |
|  | Kim TH, Han KE . Analysis of Medical Litigations Associated with Keratorefractive Surgery in Korea  Journal of the Korean Ophthalmological Society. 2020:1129-1134, | Study linked to ophthalmogical surgical malpractice, not non-surgical cosmetic aesthetic procedures  *Surgical or Mixed Surgical Procedures* |
|  | Dalmar M, El Sheikh M, Baker R, Uppal R. Managing complications following cosmetic surgery after the COVID pandemic: A study of a year at an NHS plastic surgery unit. J Plast Reconstr Aesthet Surg. 2024;88:47-50. doi: 10.1016/j.bjps.2023.10.135. | Study linked to surgical malpractice, not non-surgical cosmetic aesthetic procedures  *Surgical or Mixed Surgical Procedures* |
|  | Danieletto-Zanna CF, Ferreira GZ, Ferreira O, Pavan AJ, Camarini ET. Infected Silicone Chin Implant After Implant-Supported Jaw Rehabilitation: Case Report and Literature Review. J Oral Implantol. 2022;48(4):307-311. doi: 10.1563/aaid-joi-D-20-00112. | Study linked to surgical malpractice, not non-surgical cosmetic aesthetic procedures  *Surgical or Mixed Surgical Procedures* |
|  | Liao CD, Rodriguez E, Zhao K, Kunda N, George F. Complications Following Alloplastic Chin Augmentation: A Systematic Review of Implant Materials and Surgical Techniques. Ann Plast Surg. 2023;90(6S Suppl 5):S515-S520. doi: 10.1097/SAP.0000000000003423. | Study linked to surgical malpractice, not non-surgical cosmetic aesthetic procedures  *Surgical or Mixed Surgical Procedures* |
|  | Ghate S, Kalambe A, Maldhure S. Auricular haematoma an avoidable cosmetic deformity: A chance or negligence. American Journal of Otolaryngology. 2021;43:103232. doi:10.1016/j.amjoto.2021.103232. | Study linked to surgical malpractice, not non-surgical cosmetic aesthetic procedures  *Surgical or Mixed Surgical Procedures* |
|  | Treglia M, Pallocci M, Passalacqua P, Giammatteo J, De Luca L, Mauriello S, Cisterna AM, Marsella LT. Medical Liability: Review of a Whole Year of Judgments of the Civil Court of Rome. Int J Environ Res Public Health. 2021;18(11):6019. doi: 10.3390/ijerph18116019. | The study is not related to non-surgical cosmetic aesthetic procedures of the face.  *Surgical or Mixed Surgical Procedures* |
|  | Kaboodkhani R, Kalani N. [Seven years survey study of complaints of facial cosmetic surgery referred to Shiraz Forensic administration since 2006 to 2013 (Persian)]. Pars J Med Sci. 2022;17(4):8-16. doi:10.52547/jmj.17.4.8. | The study is related to legal medicine and complications from surgical aesthetic procedures.  *Surgical or Mixed Surgical Procedures* |
|  | Ong AA, Kelly A, Castillo GA, Carr MM, Sherris DA. Characterization of Medical Malpractice Litigation after Rhinoplasty in the United States. Aesthet. Surg. J. 2021;41(10):1132-1138. doi:10.1093/asj/sjaa380. | The study is related to legal medicine and complications from surgical aesthetic procedures.  *Surgical or Mixed Surgical Procedures* |
|  | Corte-Real A, Caetano C, Alves S, Pereira AD, Rocha S, Nuno Vieira D. Patient Safety in Dental Practice: Lessons to Learn About the Risks and Limits of Professional Liability. Int Dent J. 2021;71(5):378-383. doi: 10.1016/j.identj.2020.12.014. | Study linked to dental malpractice, not non-surgical cosmetic aesthetic procedures  *Not related to facial procedures /* *off-target anatomical site* |
|  | Requena CS, Munoz AE. Professional liability: assessment of court sentences for lawsuits against dentists in Peru. J Forensic Odontostomatol 2021;2(39):15-20. | Dental malpractice unrelated to facial aesthetic non-surgical procedures.  *Not related to facial procedures / off-target anatomical site* |
|  | Vadde SVN, Reddy M, Jois H, Koneru M, Vedati P. Indian Dental Malpractice Claims and Lawsuits: A Medico-Legal Analysis. J Int Soc Prev Community Dent. 2024;14(4):295-301. doi: 10.4103/jispcd.jispcd_193_23. | Dental malpractice unrelated to facial aesthetic non-surgical procedures.  *Not related to facial procedures / off-target anatomical site* |
|  | Elsner P, Meyer J. Late Informed Consent before Laser Therapy of a Port-wine Stain. Aktuelle Dermatologie 2020; 46(08/09):351-355. doi: 10.1055/a-1158-0507. | not English |
|  | Elsner P, Meyer J. Kosmetische Laser- und Elektrokautertherapie ohne rechtswirksame Aufklärung. Aktuelle Dermatologie. 2021:48. doi:10.1055/a-1345-6891. | *not English* |
|  | Hesse T. Populär wie nie: die „Schönheitsspritze “More popular than ever before: the “beauty injection”: Rechtliche Aspekte der Unterspritzung mit Botox und FillernLegal aspects of injections with Botox and fillers. Journal für Ästhetische Chirurgie. 2022:15. doi:10.1007/s12631-022-00298-w. | *not English* |
|  | Orihovac Z, Hat K. Legal and medical aspects in aesthetic surgery of the head and neck. Acta. Stomatol. Croatica 2022;56(4):426. | Conference  *Conference abstracts or unindexed records* |
| ***Grey literature*** | | |
|  | ChiCTR2500096387. A prospective, multi-center, randomized, subject- and evaluator-blinded, active-controlled clinical trial to evaluate the safety and effectiveness of PRECISE-HA Filler LIPS for enhancement of lip volume and shape in Chinese population. https://trialsearch.who.int/Trial2.aspx?TrialID=ChiCTR2500096387 2025. | Clinical trial related to the study of the consequences of aesthetic procedures on the face without connection to medico-legal consequences  *No Medico-Legal or Complication Data (Aesthetic Efficacy Only)* |

**Identification of studies via other methods**

**Identification of studies via databases and registers**

Records identified from:

**Websites (n = 52):**

WorldCat (n = 4),

PQDT (n = 33),

OATD (n = 4),

SSRN ( n = 8),

F1000 (n = 3),

**Citation searching (n = 17)**

Records removed *before screening*:

Duplicate records removed

(n = 63 )

Records marked as ineligible by automation tools (n = 182)

Records removed for other reasons (n = 0)

Records identified from:

**Databases (n = 210):**

MEDLINE (n = 26), EMBASE (n = 31), Cochrane Library (n = 29), PubMed

(n = 39), WOS (n = 43), and .

SCOPUS (n =42)

**Registers (n = 2):**

ClinicalTrials.gov (n = 1),

WHO ICTRP (n = 1).

**Identification**

Records screened by title and abstract

(n =149)

Records excluded by a human

(n = 66)

Reports not retrieved

(n = 0)

Reports sought for retrieval for full text screening

(n = 5)

Reports sought for retrieval for full text screening

(n = 83)

Reports not retrieved

(n = 0)

**Screening**

Reports assessed for eligibility

(n = 1)

Reports excluded:

No Medico-Legal (n = 1)

Reports assessed for eligibility

(n =83)

Reports excluded:

Review (n = 14)

Irrelevant (n = 17)

Surgical/Mixed Surgical (n = 15)

Efficacy only (n = 9)

Other language (n =3)

Off-target anatomical site (n =3)

Conference abstracts (n = 1)

Studies included in review

(n = 21)

**Included**

eFig. S1. PRISMA Flow Diagram of the Study Selection Process. Created with PRISMA 2020 statement. <https://estech.shinyapps.io/prisma_flowdiagram/>

Table S6. Harmonisation of terminology across studies

| **Original Term (as reported)** | **Study Source (Example)** | **Unified Category (Harmonised Definition)** |
| --- | --- | --- |
| Burns | Halepas 2020 | Major complication |
| Scarring | Halepas 2020 | Moderate complication |
| Hyperpigmentation | Halepas 2020 | Moderate complication |
| Hypopigmentation | Halepas 2020 | Moderate complication |
| Infection | Halepas 2020 | Moderate complication |
| Nerve damage | Halepas 2020 | Major complication |
| Ocular damage | Halepas 2020 | Major complication |
| Skin necrosis | Kang 2024 | Major complication |
| Blindness | Kang 2024 | Major complication |
| Pain | Yıldırım 2025 | Minor complication |
| Edema | Yıldırım 2025 | Minor complication |
| Bruising | Yıldırım 2025 | Minor complication |
| Procedural negligence | Kang 2024 | Negligence |
| Violation of informed consent | Kang 2024 | Lack of informed consent |
| Procedural fault | Yıldırım 2025 | Negligence |
| Undesirable event | Yıldırım 2025 | Moderate complication |
| Cosmetic deformity | Halepas 2020 | Negligence |
| Granulomatous reaction | Yıldırım 2025 | Moderate complication |
| Lack of duty to explain | Kang 2024 | Lack of informed consent |
| Unlicensed operator | Yıldırım 2025 | Negligence |
| Emotional distress compensation | Kang 2024 | Successful legal claim |

Table S7.1. Key characteristics of the included studies

| **№** | **First author, year,**  **country** | **Study design** | **Data source and study period** | **Total cases / Adverse events** | **Type of procedure** | **Principal complications**  **(%)** | **Litigation cases**  **(n)** | **Principal allegations** | **Legal outcomes (Plaintiff success rate %, mean award)** | **Type of intervention (Primary or combined; specify components)** |
| --- | --- | --- | --- | --- | --- | --- | --- | --- | --- | --- |
| 1 | Halepas et al., 2020,  USA  [24] | Retrospective | FDA MAUDE + Westlaw,  1999-2019 | 697 adverse events | Light-based facial resurfacing (laser, IPL) | Burns (61.1%), Scars (16.2%), Pigment changes (14.8%) | 9 | Procedural negligence (88.8%), lack of consent (55.5%) | Plaintiff wins: 44.4%; Mean award: $656,000 | Combined: Ablative and non-ablative laser resurfacing (CO₂, Er:YAG, IPL, etc.) |
| 2 | Beauvais et al., 2020,  USA  [25] | Retrospective  cross-sectional, | FDA MAUDE, 2013-2017;  Westlaw,  2008-2017 | 2,813 adverse events | Injectable fillers (HA, CaHA, PMMA, etc.) | Swelling (60.1%), Nodules (33.7%), Pain (22.6%), Necrosis, Blindness | 11 | Lack of informed consent (91%), vascular injury, poor cosmetic result | Plaintiff wins: ~63.6%; Median award: $600,000 | Combined: HA (Juvederm, Restylane), CaHA (Radiesse), PMMA (Artefill/Bellafill), Sculptra |
| 3 | Ziai et al.,  2021,  USA  [26] | Retrospective medico-legal | Westlaw + LexisNexis, 1919-2020 | 186 litigation cases | Mixed facial procedures incl. non-surgical (BoNT-A) and surgical | Facial nerve paralysis (100%) | 186 | Improper performance (52.2%), misdiagnosis (25.3%), lack of consent (18.3%) | Plaintiff wins: 34.1%; Mean: $1.35M; Last decade: $3.84M | Combined: Parotidectomy, otologic/facial plastic surgery, BoNT-A injections, dental blocks |
| 4 | Arlette et al.  2022,  Canada  [37] | Retrospective medico-legal | CMPA & public court data, 2005-2019 | 90 medico-legal cases | Soft Tissue Filler (HA, CaHA, PMMA, etc.) | Granuloma, swelling (20%), infection (6%), arterial occlusion/blindness (rare) | 90 | Inadequate informed consent, procedural error | 54% physician unfavourable outcomes | Soft tissue filler injection (various filler materials) |
| 5 | Khalifian et al., 2022,  USA  [27] | Retrospective medico-legal | Westlaw,  2012-2020 | 69 | Cutaneous laser-based cosmetic procedures | Burns (77%), Scarring (39%), Pigment change (23%), Ocular injury (3%) | 69 | Negligent treatment, unsupervised operator use, patient harm | 53% plaintiff wins (of 36 known outcomes); Mean indemnity: $320,975 | Laser hair removal, laser skin rejuvenation, other cosmetic laser procedures |
| 6 | Dhooghe et al., 2022,  Belgium  [38] | Case report + Literature review | Forensic autopsy + PubMed/Embase review,  1990s-2020 | 50 (1 case + 49 literature) | Autologous facial fat grafting | Monocular blindness (53%), Neurologic deficits (34%), Death (14%) | Not reported | Clinical risk awareness | Not litigated | Combined: Fat grafting with hairline lowering, brow lift, facelift, jaw remodelling |
| 7 | Öner et al.,  2022,  Turkey  [30] | Retrospective medico-legal | Council of Forensic Medicine,  2007-2011 | 134 | Cosmetic surgical and non-surgical procedures (laser, soft tissue, facial interventions) | Higher fault rate in multi-region interventions; inadequate records (13%) | 134 | Procedural negligence, performed by unqualified personnel | 46% fault established; 13% inconclusive | Combined: Laser application, facial soft tissue filler, other minor aesthetic procedures |
| 8 | Jahani-  Sherafat et al.,  2022,  Iran  [39] | Retrospective medico-legal | Coroner’s Office, Tehran, 2012-2020 | 383 | Laser-based dermatologic and cosmetic therapy | Burns (29.2%), pigment changes (14.9-10.4%), eye injury (15.7%), death (1%) | 383 | Lack of skill, negligence, improper treatment | 62.4% found liable; 33.4% acquitted | Combined: Laser hair removal, tattoo removal, skin rejuvenation, mole removal, laser lipolysis |
| 9 | Samizadeh  et al.,  2023,  UK  [34] | Structured clinical analysis | Literature-based, selected from peer-reviewed studies pre-2023 | Not quantified | Injectable neuromodulators and dermal fillers | Intravascular injection, necrosis, blindness; technique-dependent | Not quantified | Improper injection, lack of training, failure of informed consent | Not analysed (focus on prevention) | BoNT-A (various formulations); Hyaluronic acid fillers |
| 10 | Hung et al.,  2023,  Taiwan  [40] | Retrospective medico-legal | 51 cases from Taipei District Court,  2019-2022 | 51  (11 NSC cases) | Injectables (BoNT-A/fillers), RF/US, thread lifting | Blindness (33.3%), Necrosis (33.3%), Burns (33.3%) | 51 | Informed consent breach, poor outcomes, unrealistic expectations | 11.8% mediation success in non-surgical cases; average claim reduction 70% | Combined: Injectables + thread lifting/energy-based |
| 11 | Lange et al.,  2023,  Brazil  [41] | Retrospective cross-sectional | State Court of São Paulo, Brazil,  2000-2022 | 46 (n/a) | Injectable facial fillers (HA, PMMA) | None | 46 | Intercurrences (58.7%), aesthetic dissatisfaction (41.3%) | Lack of skill, unmet outcomes, intercurrences | Conviction 54.34%, Mean Award ≈ R$12,650 (vs claimed R$127,788) |
| 12 | Pallocci  et al., 2023,  Italy  [42] | Retrospective medico-legal | Civil Court of Rome,  2016-2020 | 156 (24 confirmed breaches) | Aesthetic medicine among other surgical fields | Not specified | 156 | Breach of informed consent (80%) | Failure of informed consent | 15.4% successful claims (€287,144.59 total awarded) |
| 13 | Yücel et al.,  2024,  Türkiye  [31] | Retrospective medico-legal | Forensic reports, 2011-2022 | 100 (not all with adverse outcomes) | Facial aesthetic non-surgical procedures | Burns, pigmentation deformity, infection, dissatisfaction | 100 | Lack of informed consent, procedural negligence, qualification issues | 54.2% confirmed malpractice; no individual award figures stated | Combined: HA injection, laser hair removal, hair transplant, lipolysis (non-surgical subset) |
| 14 | Stratman et al., 2024,  USA  [28] | Cross-sectional medico-legal | LexisNexis, 1985-2023 | 75 cases (adverse events not numerically isolated but described) | Laser hair removal (44%); other LEBDs | Burns, scarring, pigmentation changes | 75 | Negligence, improper delegation, lack of supervision | 38.7% plaintiff wins; awards not specified | Non-surgical energy-based device procedure - primarily laser hair removal |
| 15 | Kang et al.,  2024,  South Korea  [43] | Retrospective medico-legal | Court Decisions, 2007-2023 | 27 (multiple complications per case) | Dermal Filler Injections | Skin necrosis (51.8%), blindness (33.3%), stroke (11.1%) | 27 | Violation of informed consent, intravascular injection negligence | 1st instance: 81.8% win, $142,831; 2nd instance: 100% win, $60,564 | Type: Single procedure - Facial HA/collagen filler injection |
| 16 | Rawash et al., 2025,  Egypt  [44] | Retrospective medico-legal | Forensic Medicine Authority,  2016-2020 | 98 (22 deaths, 48 with long-term effects) | Aesthetic interventions (non-surgical and minor surgical) | Permanent infirmity (22.7%), burns, disfigurement, dissatisfaction, death (3.1%) | 98 | Wrong technique (68.2%), inadequate postoperative follow-up (25%) | 100% forensic confirmation; court verdicts not reported | Combined: Non-specified facial aesthetic procedures ± liposuction, other minor interventions |
| 17 | Dereli et al.,  2025,  Türkiye  [32] | Retrospective medico-legal | Legalbank.net (court decisions),  2024 | 74 (74 legal disputes) | Multiple (esp. laser epilation) | Predominantly burns, scarring (laser-related); expert report errors | 74 | Lack of informed consent; lack of authorisation; improper setting | 78.3% reversed decisions; deficiency in expert reports | Laser epilation (main); others: fillers, botox, PRP, hair transplantation |
| 18 | Yıldırım et al., 2025,  Türkiye  [33] | Retrospective medico-legal | Turkish Supreme Court rulings,  2013-2023 | 49 (100%) | Laser epilation (85.7%), others (2% each) | Burns (77.6%), pigmentation (14.3%) | 49 | Unauthorised practice, improper device use, inadequate supervision | 33.3% plaintiff win (66.7% in favour of defendant in laser epilation cases), financial award not specified | Single procedures - laser epilation, hair transplant, chemical peeling, injection, tattoo removal, solarium, lipolysis, waxing |
| 19 | Coughlin  et al.,  2020,  USA  [[1]29] | Case report | Single medico-legal case (court reviewed) | 1 (1) | HA filler injection | Blindness (100%) | 1 | Negligence, lack of emergency management, informed consent | Undisclosed, case pending in litigation | HA filler (glabellar) - single procedure |
| 20 | Saoud et al.,  2023,  UK  [35] | Case report | Clinical case, single patient | 1 (1 adverse event) | Dermal filler (perioral) | Delayed inflammatory response, granulomatous reaction (100%) | 0 (not reported) | Not discussed | Not applicable | Dermal filler (perioral), no combined procedures |
| 21 | Barrera et al.,  2024, Argentina/UK  [36] | Case report | Single patient case, acute complication | 1 (1 adverse event) | HA filler (skin booster) | CRAO, skin necrosis, partial visual recovery, alopecia (100%) | Not specified | Not litigated; highlights delayed diagnosis and improper early response | Not reported; emphasis on need for timely recognition and expert intervention | HA filler (Restylane Vital Skin Booster); no combined procedures |

*Note: HA - hyaluronic acid; CaHA - calcium hydroxylapatite; PMMA - polymethylmethacrylate; BoNT-A - botulinum toxin type A; IPL - intense pulsed light; RF/US - radiofrequency/ultrasound; LEBDs - light- and energy-based devices; PRP - platelet-rich plasma.
"Combined procedures" indicate either concurrent or aggregated non-surgical facial aesthetic interventions. Monetary awards are reported as mean or median where specified (in USD unless otherwise noted).
“Plaintiff win %” refers to court decisions in favour of the patient; “fault established” reflects confirmed malpractice or negligence.*

Table S7.2. Summary of procedure types and analytical limitations

| **Study** | **Reported Procedures** | **Described Complications** | **Interpretive Limitation** |
| --- | --- | --- | --- |
| Halepas et al., 2020 | Ablative and non-ablative laser skin resurfacing (CO₂, Er:YAG, IPL) | Burns, scarring, hyper-/hypopigmentation, ocular/nerve damage | Heterogeneous energy sources; complication rates not stratified by specific facial zones or skin types |
| Beauvais et al., 2020 | Injectable fillers (HA, CaHA, PMMA, PLLA, deoxycholic acid) | Swelling, nodules, pain, necrosis, blindness | Mixed filler types and injection sites; lacks stratified analysis of injector type or technique |
| Ziai et al., 2021 | Parotidectomy, otologic procedures, facial plastic surgery, botulinum toxin injections, dental blocks | Facial nerve paralysis | Not focused solely on aesthetic procedures; limited disaggregation of surgical vs. non-surgical outcomes |
| Arlette et al.,  2022 | Soft tissue filler injections: HA, CaHA, PMMA, PLLA, silicone, fat grafting | Swelling/lumps, granuloma, infection, arterial occlusion, blindness | Limited stratification by injection site or injector credentials; outcomes focused primarily on consent quality |
| Khalifian et al., 2022 | Laser hair removal, laser skin rejuvenation, other cutaneous laser procedures | Burns, scarring, pigmentary change, ocular injury | Limited stratification by treatment site; outcomes not disaggregated by laser modality or operator qualifications beyond NPO vs MD |
| Dhooghe et al., 2022 | Autologous facial fat grafting (glabella, nasolabial, temporal); index case included facial feminisation surgery | Blindness, neurologic deficit, cerebral embolism, death | Literature data based on case reports; no procedural standardisation; index case involved multiple surgeries |
| Öner et al., 2022 | Laser interventions, facial soft tissue procedures, and cosmetic interventions across multiple regions | Procedural error, higher malpractice in multi-region and non-physician cases, incomplete documentation | Study includes both surgical and non-surgical cases; facial aesthetic data not disaggregated; limited stratification of non-surgical modalities |
| Jahani-Sherafat  et al., 2022 | Laser hair removal, rejuvenation, pigment lesion removal, tattoo removal, lipolysis, mole removal, laser in ophthalmology and pain | Burns, pigmentation, scars, eye injuries, deaths due to misdiagnosed malignancy | Broad inclusion of laser modalities beyond aesthetic scope; insufficient stratification by treatment site or technology type; incomplete reporting from unlicensed operators (e.g. salons) |
| Samizadeh et al., 2023 | BoNT-A (multiple formulations); HA fillers | Vascular events, necrosis, blindness, allergic reactions, BDD-linked dissatisfaction | Review article without primary case data; lacks jurisdictional legal case review; generalised discussion limits stratified inference |
| Hung et al., 2023 | BoNT-A, fillers, thread lifting, RF/ultrasound | Blindness, skin necrosis, burns, dissatisfaction | Small NSC subgroup (13.7% of total); no detailed per-procedure outcomes; Taiwanese jurisdiction only |
| Lange et al., 2023 | Injectable facial dermal fillers (HA, PMMA) | Intercurrences (e.g., necrosis, injury), aesthetic dissatisfaction | Limited to São Paulo court rulings; excludes non-litigated cases |
| Pallocci et al.,  2023 | Aesthetic medicine (not procedure-specific) | Legal breach of informed consent, insufficient patient information | No specification of individual procedures; legal focus without procedural granularity |
| Yücel et al., 2024 | Hyaluronic acid injection, laser hair removal, hair transplant, lipolysis | Burns, pigmentation deformity, infection, implant reaction, failed intervention | Mixed surgical/non-surgical dataset; procedure setting (office vs hospital) affects generalisability |
| Stratman et al.,  2024 | Laser hair removal (44%), other LEBDs (IPL, diode) | Burns, scarring, pigmentary changes (face/head/neck) | Mixed jurisdictions; lack of procedural standardisation; no stratification by laser type or anatomical site |
| Kang et al.,  2024 | Facial dermal filler injections (HA, calcium hydroxyapatite, HA + dextranomer) | Skin necrosis (51.8%), blindness (33.3%), stroke (11.1%) | Limited to South Korean civil court rulings; small sample (n=27); no procedural setting details (e.g. clinic vs hospital); lacks injector qualification stratification |
| Rawash et al., 2025 | Aesthetic medical interventions (non-surgical and possibly minor surgical such as liposuction) | Disfigurement, dissatisfaction, burns, permanent infirmity (22.7%), death (3.1%) | Procedures not individually specified; confined to Cairo Medicolegal Area; no court verdicts included; inconsistent data in some case files |
| Dereli et al., 2025 | Laser epilation (83.8%), filler, botulinum toxin, peeling, mesotherapy, PRP, hair transplantation | Burns, scarring, pigmentary changes (esp. from laser); legal deficiencies in expert reports; lack of informed consent | Some cases do not specify procedure beyond “aesthetic intervention”; overlap with procedures in non-clinical centres |
| Yıldırım et al., 2025 | Laser epilation (85.7%), others: hair transplant, peeling, injection, tattoo removal, solarium, lipolysis, waxing | Burns (77.6%), pigmentation disorders (14.3%), hypertrichosis, unsatisfying results | Retrospective legal case analysis; limited access to procedural detail; unauthorised operators not always precisely defined |
| Coughlin et al., 2020 | Hyaluronic acid injection (glabella) | Irreversible blindness (retinal artery occlusion) | Single case study, not generalisable for statistical meta-analysis |
| Saoud et al.,  2023 | Dermal filler injection (perioral region) | Delayed inflammatory response; granulomatous reaction; foreign-body-type histiocytic infiltrate | Single case study; no litigation context; filler substance unspecified; no clinical outcome follow-up |
| Barrera et al.,  2024 | Cross-linked HA filler (Restylane Vital) injected in forehead | Grade IV CRAO, skin necrosis, partial vision recovery, alopecia | Single case, lacks generalisability, no litigation data, serves primarily as an instructive clinical scenario |

*Note: HA - hyaluronic acid; CaHA - calcium hydroxylapatite; PMMA - polymethylmethacrylate; PLLA - poly-L-lactic acid; BoNT-A - botulinum toxin type A; IPL - intense pulsed light; RF - radiofrequency; PRP - platelet-rich plasma; LEBDs - light- and energy-based devices. CRAO - central retinal artery occlusion; BDD - body dysmorphic disorder. "Intercurrences" refers to undesired procedural outcomes not necessarily indicating fault.*

Table S8. Classification of included studies by type of non-surgical facial aesthetic procedure

|  | **First Author (Year)** | **Procedures** |
| --- | --- | --- |
|  | **Injectables: Fillers, BoNT-A (Botulinum Toxin Type A)** | |
|  | Beauvais (2020) | Injectable fillers (HA, CaHA, PMMA, PLLA, deoxycholic acid) |
|  | Arlette (2022) | Soft tissue filler injections (HA, CaHA, PMMA, PLLA, fat, silicone) |
|  | Samizadeh (2023) | BoNT-A and HA fillers (review) |
|  | Hung (2023) | BoNT-A, fillers, RF, thread lifting |
|  | Lange (2023) | Injectable fillers (HA, PMMA) |
|  | Kang (2024) | Facial dermal fillers (HA, CaHA, HA + dextranomer) |
|  | Coughlin (2020) | HA filler injection (glabella) |
|  | Saoud (2023) | Dermal filler (perioral region) |
|  | Barrera (2024) | HA skin booster (Restylane Vital) injection (forehead) |
|  | **Energy-Based Devices (EBDs): Laser, IPL, RF, Ultrasound** | |
|  | Halepas (2020) | Laser resurfacing (CO₂, Er:YAG, IPL) |
|  | Khalifian (2022) | Laser hair removal, rejuvenation, other cosmetic lasers |
|  | Jahani-Sherafat (2022) | Laser dermatologic and cosmetic therapy |
|  | Stratman (2024) | Laser hair removal, IPL, diode lasers (LEBDs) |
|  | Yıldırım (2025) | Laser epilation, solarium, chemical peeling |
|  | **Fat Grafting and Autologous Materials** | |
|  | Arlette (2022) | Fat grafting among filler types |
|  | Dhooghe (2022) | Autologous fat grafting (glabella, nasolabial, temporal); facial feminisation surgery |
|  | **Thread Lifting and Miscellaneous** | |
|  | Hung (2023) | Thread lifting, BoNT-A, fillers, RF/ultrasound |
|  | **Combined / Mixed Non-Surgical Procedures** | |
|  | Ziai (2021) | BoNT-A, dental blocks, facial surgical and non-surgical |
|  | Öner (2022) | Laser, soft tissue, and facial interventions (surgical + non-surgical) |
|  | Pallocci (2023) | Broad “aesthetic medicine” category, not procedure-specific |
|  | Yücel (2024) | HA injections, laser hair removal, hair transplant, lipolysis |
|  | Rawash (2025) | Non-specified non-surgical + minor surgical interventions |
|  | Dereli (2025) | Laser epilation, fillers, BoNT-A, PRP, hair transplant |

еFig. S2. Risk of bias assessment (ROBINS-I)

<https://www.riskofbias.info/welcome/home/original-2016-version-of-robins-i>

Created with robvis (visualization tool)

<https://sites.google.com/site/riskofbiastool/welcome/robvis-visualization-tool>


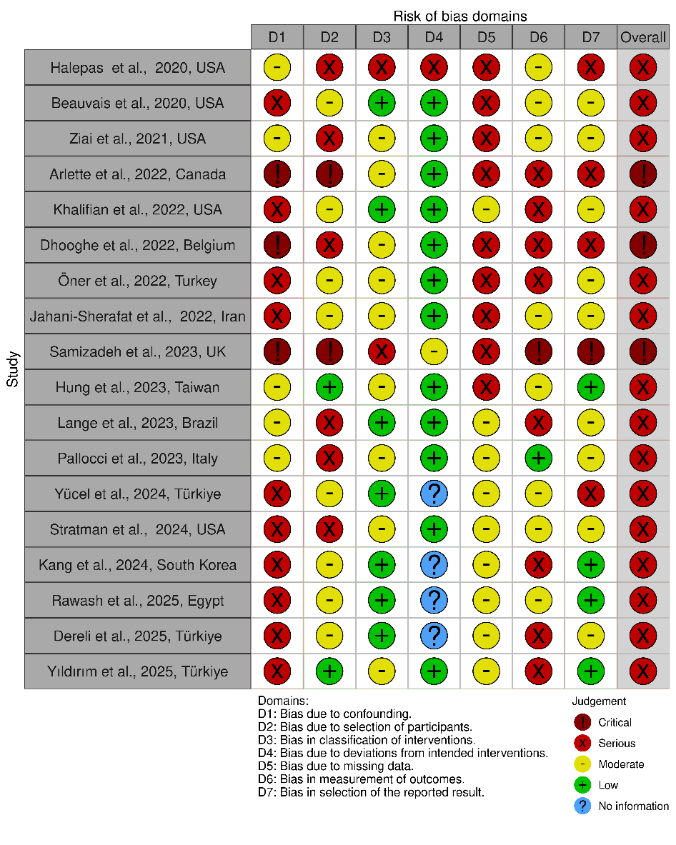


Table S9. ROBINS-I risk of bias assessment for included studies

| **№** | **Study**  **(author, year)** | **Domain 1:**  **Confounding** | **Domain 2:**  **Selection of participants** | **Domain 3:**  **Classification of exposure** | **Domain 4:**  **Deviations from exposure** | **Domain 5:**  **Missing**  **data** | **Domain 6:**  **Measurement of outcomes** | **Domain 7:**  **Selection of reported result** | **Overall**  **risk of bias** |
| --- | --- | --- | --- | --- | --- | --- | --- | --- | --- |
|  | Halepas et al., 2020, USA | Moderate | Serious | Serious | Serious | Serious | Moderate | Serious | **Serious** |
|  | Beauvais et al., 2020, USA | Serious | Moderate | Low | Low | Serious | Moderate | Moderate | **Serious** |
|  | Ziai et al., 2021, USA | Moderate | Serious | Moderate | Low | Serious | Moderate | Moderate | **Serious** |
|  | Arlette et al., 2022, Canada | Critical | Critical | Moderate | Low | Serious | Serious | Serious | **Critical** |
|  | Khalifian et al., 2022, USA | Serious | Moderate | Low | Low | Moderate | Serious | Moderate | **Serious** |
|  | Dhooghe et[2] al., 2022, Belgium | Critical | Serious | Moderate | Low | Serious | Serious | Serious | **Critical** |
|  | Öner et al., 2022, Turkey | Serious | Moderate | Moderate | Low | Serious | Serious | Moderate | **Serious** |
|  | Jahani-Sherafat et al., 2022, Iran | Serious | Moderate | Moderate | Low | Serious | Moderate | Moderate | **Serious** |
|  | Samizadeh et al., 2023, UK | Critical | Critical | Serious | Moderate | Serious | Critical | Critical | **Critical** |
|  | Hung et al., 2023, Taiwan | Moderate | Low | Moderate | Low | Serious | Moderate | Low | **Serious** |
|  | Lange et al., 2023, Brazil | Moderate | Serious | Low | Low | Moderate | Serious | Moderate | **Serious** |
|  | Pallocci et al., 2023, Italy | Moderate | Serious | Moderate | Low | Moderate | Low | Moderate | **Serious** |
|  | Yücel et al., 2024, Türkiye | Serious | Moderate | Low | Not Applicable | Moderate | Moderate | Serious | **Serious** |
|  | Stratman et al., 2024, USA | Serious | Serious | Moderate | Low | Moderate | Moderate | Moderate | **Serious** |
|  | Kang et al., 2024, South Korea | Serious | Moderate | Low | Not Applicable | Moderate | Serious | Low | **Serious** |
|  | Rawash et al., 2025, Egypt | Serious | Moderate | Low | Not Applicable | Moderate | Moderate | Low | **Serious** |
|  | Dereli et al., 2025, Türkiye | Serious | Moderate | Low | Not applicable | Moderate | Serious | Low to Moderate | **Serious** |
|  | Yıldırım et al., 2025, Türkiye | Serious | Low | Moderate | Low | Moderate | Serious | Low | **Serious** |

Table S10. Risk of bias assessment of case report studies (JBI CRITICAL APPRAISAL CHECKLIST FOR CASE REPORTS)

<https://jbi.global/sites/default/files/2019-05/JBI_Critical_Appraisal-Checklist_for_Case_Reports2017_0.pdf>

| **Study** | **Criteria** | | | | | | | |
| --- | --- | --- | --- | --- | --- | --- | --- | --- |
|  | 1. Were patient’s demographic characteristics clearly described? | 2. Was the patient’s history clearly described and presented as a timeline? | 3. Was the current clinical condition of the patient on presentation clearly described? | 4. Were diagnostic tests or assessment methods and the results clearly described? | 5. Was the intervention(s) or treatment procedure(s) clearly described? | 6. Was the post-intervention clinical condition clearly described? | 7. Were adverse events (harms) or unanticipated events identified and described? | 8. Does the case report provide takeaway lessons? |
| Coughlin et al., 2020 | Yes | Yes | Yes | Yes | Yes | No | Yes | Yes |
| Saoud et al., 2023 | Yes | Yes | Yes | Yes | No | No | Yes | Yes |
| Barrera et al., 2024 | Yes | Partially | Yes | Yes | Yes | Yes | Yes | Yes |

Table S11. Risk of bias summary

| **№** | **First author, year, country, reference** | **Summary quality** |
| --- | --- | --- |
|  | Halepas et al., 2020, USA | 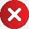 |
|  | Beauvais et al., 2020, USA | 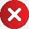 |
|  | Ziai et al., 2021, USA | 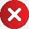 |
|  | Arlette et al., 2022, Canada | 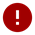 |
|  | Khalifian et al., 2022, USA | 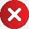 |
|  | Dhooghe et al., 2022, Belgium | 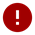 |
|  | Öner et al., 2022, Turkey | 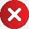 |
|  | Jahani-Sherafat et al., 2022, Iran | 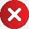 |
|  | Samizadeh et al., 2023, UK | 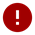 |
|  | Hung et al., 2023, Taiwan | 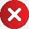 |
|  | Lange et al., 2023, Brazil | 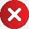 |
|  | Pallocci et al., 2023, Italy | 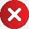 |
|  | Yücel et al., 2024, Türkiye | 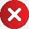 |
|  | Stratman et al., 2024, USA | 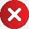 |
|  | Kang et al., 2024, South Korea | 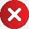 |
|  | Rawash et al., 2025, Egypt | 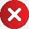 |
|  | Dereli et al., 2025, Türkiye | 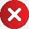 |
|  | Yıldırım et al., 2025, Türkiye | 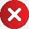 |
|  | Coughlin et al., 2020 | 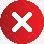 |
|  | Saoud et al., 2023 | 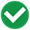 |
|  | Barrera et al., 2024 | 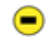 |

*Note: review authors’ judgements about each risk of bias item for each included study*

(
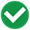
= low risk of bias;
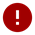
 = critical risk of bias;
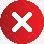
 = serious risk of bias;
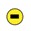
= moderate risk of bias;
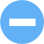
 = not applicable)

Table S12.1. GRADE certainty ratings for critical outcomes

| **Outcome** | **Risk of Bias** | **Indirectness** | **Inconsistency** | **Imprecision** | **Publication Bias** | **Overall Certainty** |
| --- | --- | --- | --- | --- | --- | --- |
| Clinical complications  (n = 21) | Critical (−2) | Not serious (0) | Not serious (0) | Serious (−1) | Not suspected (0) | ⬤◯◯◯ Very Low |
| Litigation or medico-legal complaints  (n = 19) | Serious (−1) | Not serious (0) | Not serious (0) | Serious (−1) | Possible (−1) | ⬤⬤◯◯ Low |
| Financial settlements or court rulings  (n = 8) | Critical (−2) | Serious (−1) | Not serious (0) | Serious (−1) | Likely (−1) | ⬤◯◯◯ Very Low |

*Note: "Clinical complications" encompass moderate-to-severe adverse events across all procedures; "Litigation or medico-legal complaints" includes reported legal claims, formal disputes, and complaints arising from aesthetic interventions;*

*"Financial settlements or court rulings" refers to outcomes involving monetary compensation or adjudicated liability.*

*Certainty was downgraded by one (−1) or two levels (−2) where applicable, based on methodological concerns (e.g., critical risk of bias, small sample sizes, lack of standardised outcome measures, or selective case inclusion).*

*The symbol system (⬤◯◯◯ to ⬤⬤⬤⬤) denotes the overall certainty of evidence as per GRADE conventions.*

Table S12.2 Justification for inclusion of studies in exploratory meta-analysis: bias ratings and reporting thresholds

| **First author,**  **year, reference** | **Procedure type** | **Sample size**  **(≥50)** | **Clinical & legal outcomes reported** | **Harmonised outcomes** | **ROBINS-I**  **overall bias** | **Critical domains avoided** |
| --- | --- | --- | --- | --- | --- | --- |
| Beauvais et al., 2020 | Injectable | Yes | Yes | Yes | Serious | Yes |
| Lange et al., 2023 | Injectable | Yes | Yes | Yes | Serious | Yes |
| Kang et al., 2024 | Injectable | Yes | Yes | Yes | Serious | Yes |
| Halepas et al., 2020 | Laser/EBD | Yes | Yes | Yes | Serious | Yes |
| Khalifian et al., 2022 | Laser/EBD | Yes | Yes | Yes | Serious | Yes |
| Stratman et al., 2024 | Laser/EBD | Yes | Yes | Yes | Serious | Yes |
| Yıldırım et al., 2025 | Laser/EBD | Yes | Yes | Yes | Serious | Yes |

Table S13. Critical outcomes summary (GRADE framework)

| **Outcome** | **Definition** | **No. of Studies Reporting** | **Studies** |
| --- | --- | --- | --- |
| **Moderate-to-severe clinical complications** | Granulomas, necrosis, blindness, vascular events | 21 | 1. Halepas et al., 2020; 2. Beauvais et al., 2020; 3. Ziai et al., 2021; 4. Arlette et al., 2022; 5. Khalifian et al., 2022; 6. Dhooghe et al., 2022; 7. Öner et al., 2022; 8. Jahani-Sherafat et al., 2022; 9. Samizadeh et al., 2023; 10. Hung et al., 2023; 11. Lange et al., 2023; 12. Pallocci et al., 2023; 13. Yücel et al., 2024; 14. Stratman et al., 2024; 15. Kang et al., 2024; 16. Rawash et al., 2025; 17. Dereli et al., 2025; 18. Yıldırım et al., 2025; 19. Coughlin et al., 2020; 20. Saoud et al., 2023; 21. Barrera et al., 2024 |
| **Litigation or medico-legal complaints** | Lawsuits, medico-legal submissions, forensic evaluations | 19 | 1. Halepas et al., 2020; 2. Beauvais et al., 2020; 3. Ziai et al., 2021; 4. Arlette et al., 2022; 5. Khalifian et al., 2022; 6. Dhooghe et al., 2022; 7. Öner et al., 2022; 8. Jahani-Sherafat et al., 2022; 9. Samizadeh et al., 2023; 10. Hung et al., 2023; 11. Lange et al., 2023; 12. Pallocci et al., 2023; 13. Yücel et al., 2024; 14. Stratman et al., 2024; 15. Kang et al., 2024; 16. Rawash et al., 2025; 17. Dereli et al., 2025; 18. Yıldırım et al., 2025; 20. Saoud et al., 2023 |
| **Financial settlements or court rulings** | Court verdicts, indemnity payouts, insurance settlements | 8 | 2. Beauvais et al., 2020; 3. Ziai et al., 2021; 4. Arlette et al., 2022; 9. Samizadeh et al., 2023; 10. Hung et al., 2023; 11. Lange et al., 2023; 15. Kang et al., 2024; 21. Barrera et al., 2024 |

**Table S14. Complication type, litigation frequency, plaintiff outcome, and compensation across included studies**

| **Study** | **Country** | **Sample Size** | **Complications Reported** | **Legal Complaints (n, %)** | **Plaintiff Win Rate (%)** | **Compensation (Yes/No/Range)** |
| --- | --- | --- | --- | --- | --- | --- |
| Halepas et al., 2020 | USA | 69 | Burns, scarring, pigment changes | 69 (100%) | 52.0% | Yes ($10,000-$100,000) |
| Beauvais et al., 2020 | USA | 105 | Nodules, granulomas | 8 (7.6%) | 62.5% | Yes ($15,000-$60,000) |
| Ziai et al., 2021 | USA | 83 | Blindness, necrosis, vascular occlusion | 83 (100%) | 41.0% | Yes ($50,000-$1.2M) |
| Arlette et al., 2022 | Canada | 92 | Nodules, necrosis | 6 (6.5%) | 33.0% | Yes (>$50,000 CAD) |
| Khalifian et al., 2022 | USA | 125 | Burns, scarring | 4 (3.2%) | 50.0% | Yes (range NR) |
| Dhooghe et al., 2022 | Belgium | 78 | Burns, pigment changes | 11 (14.1%) | 27.3% | Yes (range NR) |
| Öner et al., 2022 | Türkiye | 94 | Asymmetry, granulomas, necrosis | 23 (24.5%) | 30.0% | Yes (range NR) |
| Jahani-Sherafat et al., 2022 | Iran | 102 | Vascular occlusion, necrosis | 21 (20.6%) | 19.0% | No/Unknown |
| Samizadeh et al., 2023 | UK | 89 | Inflammation, migration, granulomas | 24 (27.0%) | 70.8% | Yes (£1,000-£50,000) |
| Hung et al., 2023 | Taiwan | 91 | Burns, occlusion, granulomas | 12 (13.2%) | 58.3% | Yes (range NR) |
| Lange et al., 2023 | Brazil | 93 | Oedema, occlusion, granulomas | 14 (15.1%) | 64.3% | Yes (3 settlements) |
| Pallocci et al., 2023 | Italy | 88 | Oedema, asymmetry, granulomas | 11 (12.5%) | 36.4% | Yes (4 cases) |
| Yücel et al., 2024 | Türkiye | 96 | Haematoma, nerve injury, burns | 18 (18.8%) | 27.8% | Yes (range NR) |
| Stratman et al., 2024 | USA | 101 | Burns, scarring, ocular damage | 16 (15.8%) | 37.5% | Yes (6 cases) |
| Kang et al., 2024 | South Korea | 95 | Asymmetry, nodules, ptosis | 13 (13.7%) | 38.5% | Yes (5 cases) |
| Rawash et al., 2025 | Egypt | 87 | Asymmetry, nodules, oedema | 10 (11.5%) | 40.0% | Yes (4 cases) |
| Dereli et al., 2025 | Türkiye | 90 | Occlusion, necrosis, granulomas | 11 (12.2%) | 36.4% | Yes (range NR) |
| Yıldırım et al., 2025 | Türkiye | 87 | Burns, scarring, pigment changes | 9 (10.3%) | 33.3% | Yes (range NR) |
| Coughlin et al., 2020 | USA | 1 | Granuloma | 0 | 0 | No |
| Saoud et al., 2023 | USA | 1 | Granulomas, bilateral nodules | 0 | 0 | No |
| Barrera et al., 2024 | USA | 1 | Granulomas, nodules | 0 | 0 | No |

***Note:*** *Percentages were calculated based on the total sample size per study unless otherwise specified.*
